# Supply, demand and use of economic evidence in vaccine policymaking in Africa: A scoping review

**DOI:** 10.1371/journal.pgph.0007080

**Published:** 2026-09-24

**Authors:** Esther Mazuba, Joseph Kazibwe, Joseph Chirwa, Alick Banda, Oliver Kaonga, Bona Chitah, Stacey Orangi, Edwine Barasa, Felix Masiye

**Affiliations:** 1 Department of Economics, University of Zambia, Lusaka, Zambia; 2 Department of Clinical Sciences, Lund University, Malmö, Sweden; 3 Department of Mathematics and Statistics, University of Zambia, Lusaka, Zambia; 4 Health Economics Research Unit, KEMRI-Wellcome Trust Research Programme, Nairobi, Kenya; 5 Center for Tropical Medicine and Global Health, Nuffield Department of Medicine, University of Oxford, Oxford, United Kingdom; 6 Department of Health Metrics Sciences, University of Washington, Seattle, Washington, United States of America; University of Michigan, UNITED STATES OF AMERICA

## Abstract

Economic evaluation offers policy makers a valuable tool for optimizing the use scarce healthcare resources. This scoping review aims to describe the existing scientific literature on economic evaluation and budget impact analysis of vaccine interventions, and the use of economic evidence in Africa. It highlights the current landscape and the challenges in using economic data to support vaccine policy on the continent. This study adapted and followed the Preferred Reporting Items for Systematic Reviews and Meta-Analysis (PRISMA) guidelines. We included peer-reviewed studies published in English between January 2000 and July 2025 that reported on cost and economic evaluations of vaccines administered to children and youth, or on the use of economic evidence for policy decision-making in Africa. A literature search was conducted across five electronic databases: Medline, Embase, Web of Science, and Scopus. The search yielded 4037 articles in total after deduction of duplication. A total of 140 articles were included in the study following title, abstract screening and full text review. The review identified a sparse but growing literature of economic evaluations related to vaccine interventions in Africa. Only a handful of countries are represented in the articles published, implying a very low production of (and likely a low demand for) economic evaluation evidence. Authorship of the articles is led mostly by researchers from institutions based outside Africa, mostly in North America and Europe. Funding of studies is mostly by Gates Foundation, Gavi, PATH, Wellcome Trust and World Health Organisation. Production and use of economic evaluation for vaccine policy in Africa have been hampered by capacity limitations in both research and policy institutions on the continent. The use of the evidence in decision making is limited. In the sphere of vaccine policy, economic evaluation is still a fledging enterprise requiring more investment.

## Background

Vaccination remains one of the most effective and cost-effective public health interventions, preventing an estimated three to five million deaths annually and contributing substantially to reductions in morbidity and mortality worldwide [1,2] through the prevention of 20 diseases [3]. Over the past decades, immunization programmes have dramatically reduced the burden of vaccine-preventable diseases and contributed to improvements in child survival especially in low- and middle-income countries (LMICs) [4]. Despite these gains, Africa continues to shoulder a disproportionate burden of childhood vaccine-preven

deaths (VPDs), accounting for a large share of global childhood morbidity and mortality while facing persistent challenges in achieving equitable immunization coverage [4–6].

The sub-Saharan region alone is likely to experience more than an estimated 500,000 child related deaths from VPDs annually [7] accounting for over 58% of all child deaths, mostly from pneumococcal diseases, rotavirus, measles, pertussis, tetanus and rubella [8]. Immunisation coverage in Africa remains low with one in five children aged 0–5 years not fully vaccinated. In 2021 this included 8.7 million children who did not receive a single dose of any vaccine, referred to as “zero-dose” children [9,10]. At the same time, rapid advances in vaccine development have expanded the range of vaccines, formulations, and delivery strategies available to countries. For example, with support from Global Alliance for Vaccines and Immunisation (Gavi), there were over 300 new vaccines introduced in LMICs between 2021–2024 [1]. These developments present important opportunities to improve population health but also increase the complexity of decisions regarding vaccine introduction, procurement, financing, and delivery.

The policy environment for immunization is also changing. External financing has started to decline, and donors are making explicit calls for LMICs to step up domestic financing for immunization [6]. There is a realization that sustained progress in immunization is increasingly threatened by fiscal budget constraints at both national and international levels [11]. Many African countries are transitioning from donor-supported vaccine financing towards greater domestic resource mobilization while pursuing broader universal health coverage (UHC) goals. Simultaneously, governments must balance investments in immunization with competing health priorities within constrained public budgets. Decisions about introducing new vaccines or modifying immunization programmes therefore require explicit approaches to priority-setting that consider not only disease burden and vaccine effectiveness, but also affordability, budget impact, opportunity costs, implementation feasibility, equity, and long-term sustainability [12,13]. As vaccine policy decisions become increasingly complex, economic evidence has assumed a more prominent role in supporting efficient and transparent allocation of limited health resources.

Economic evaluation provides a structured framework for comparing the costs and consequences of alternative health interventions and assessing their value for money [14]. Specific methods of economic evaluation such as costing, cost-effectiveness analysis (CEA), cost utility analysis (CUA), cost-benefit analysis (CBA) and budget impact analysis (BIA) can inform decisions regarding vaccine introduction, programme expansion, procurement strategies, and delivery approaches by quantifying the health gains and resource implications associated with alternative options [14,15]. These analyses can help decision-makers identify interventions that maximise population health while ensuring financial sustainability. Consequently, economic evaluation has become an increasingly important component of health technology assessment (HTA), priority-setting, and evidence-informed policymaking globally.

The growing importance of economic evidence has coincided with efforts to strengthen institutional mechanisms for evidence-informed vaccine decision-making. At the global level, the World Health Organization (WHO) supports evidence-based vaccine recommendations through the Strategic Advisory Group of Experts on Immunization (SAGE), while Gavi has promoted the use of evidence in decisions regarding vaccine introduction and programme financing and strengthening countries’ prioritisation of vaccines appropriate to their context as one of its strategic objectives [5]. At the national level, some countries have established National Immunization Technical Advisory Groups (NITAGs) to provide independent recommendations based on epidemiological, economic, programmatic, and contextual evidence [16,17]. In parallel, an increasing number of African countries are institutionalising Health Technology Assessment (HTA) as a mechanism to improve transparency, efficiency, and accountability in health sector priority-setting and resource allocation [18]. Together, these developments have created growing demand for high-quality, locally relevant economic evidence to support vaccine policy decisions.

However, the existence of decision-making structures does not necessarily ensure that economic evidence is generated or routinely used in policy. Studies examining evidence-informed decision-making in LMICs have shown that the influence of research depends on a broader ecosystem comprising analytical capacity, data availability, institutional arrangements, governance, funding, stakeholder engagement, and relationships between researchers and policymakers [16]. In many African settings, challenges including limited local expertise, insufficient context-specific data, dependence on externally funded research, and weak institutional linkages continue to constrain both the production and uptake of economic evidence [16]. Understanding the contribution of economic evidence to vaccine policy therefore requires consideration of three interrelated dimensions: the supply of economic evidence (what evidence is produced, where, by whom, and for which vaccines), the demand for economic evidence (the extent to which policy institutions seek and require such evidence), and the use of economic evidence (how evidence informs vaccine introduction, financing, prioritization, and implementation decisions).

Currently, little is known about how national immunization programmes prioritize new vaccine introductions and vaccine delivery strategies. A scoping review by Guillaume et al found that much of the evidence used in priority-setting regarding vaccines is dominated by burden of disease metrics such as the number of illnesses, deaths or disability-adjusted life-years averted. Other considerations in the common criteria included vaccine price and financing [12]. However, immunisation policy makers and planners in LMICs generally lack information on the costs and cost-effectiveness analysis of immunisation interventions [19]. Despite the general understanding that the health impact of most childhood interventions is strong, the cost-effectiveness of various immunization interventions in various contexts remains unclear [19,20]. A review by Anosike et al reported the cost effectiveness and budget impact of vaccines targeting any of the three diseases that is malaria, measles and meningitis in Africa found that there was limited evidence on cost effectiveness of the vaccines in Africa [21]. However, the Anosike et al study was limited to only three diseases, did not address the challenges of economic evidence generation and use in policy making in Africa. Addressing these knowledge gaps is increasingly important as African countries assume greater responsibility for financing immunization programmes, strengthen HTA and evidence-informed decision-making processes, and seek to maximise health gains from limited public resources.

We therefore conducted a scoping review to investigate the availability and breadth of the literature on the availability of economic evaluation evidence on childhood vaccines and the use of economic evidence in Africa. This review examines the breath in economic evidence, its use in decision making and gaps that should be filled. Country level estimates of cost-effectiveness or budget impact can have a meaningful impact on the policy making process when available. For example, incorporating economic evidence into the policy process may enable country immunisation programmes to evaluate the feasibility of and planning for sustainability of vaccine interventions [22].

The main aim of this paper is to describe the existing scientific literature on economic evaluation analysis of vaccine interventions, and the use of economic evidence in Africa. This study has two specific objectives: first, to synthesise the evidence regarding the cost-effectiveness of various vaccination interventions in Africa (i.e., provide a summary of findings on economic evaluation of vaccine interventions in Africa), and secondly, to describe the application of economic evidence in decision making in Africa spelling out the enablers and challenges to inform immunization policy makers and future research.

## Methods

### Protocol and registration

This study is a scoping review that adapted and followed the Preferred Reporting Items for Systematic Reviews and Meta-Analysis for Scoping Reviews (PRISMA ScR) guidelines [23]. The protocol of the review was registered with PROSPERO registration number CRD420250653516.

### Eligibility criteria

We included studies that report on cost and economic evaluations of any vaccine administered to children and youth except COVID vaccines. The vaccines targeting COVID 19 were excluded because they are currently not meant for routine administration and are majorly targeting adults. In addition, we included studies report on the use of economic evidence in decision making in any country in Africa, published in English since 2000. The study only covered the period 2000 to July 2025 with an update search done on the 11^th^ of July 2025. The study includes only relevant vaccine types that is, those currently in use or planned to be used in the near future. We included studies of all study designs except editorials, and reviews (systematic and scoping reviews).

We excluded studies that are published in other languages other than English, protocols, studies without full text versions, and or studies done outside Africa. Studies reporting on the COVID vaccine, or vaccines that are predominantly administered to adults (for example flu shots). Studies published prior to 2000 were also excluded.

### Information sources

The study search utilized electronic databases and bibliographies to identify relevant articles to include in the study. The study searched the following electronic databases Medline, Embase, Web of Science, Scopus, and EconLit Complete. The databases were selected because they are the most prominent databases with large repositories of relevant literature. Bibliographies of included studies were searched for relevant articles.

### Search strategy

The search strategy involved the use of key words that reflect the scope of the aim of the study. The key words were identified using the PICOT framework [24]. Table 1 shows the PICOT framework.

**Table 1:** PICOT framework for the study.

Element	Description
**P**opulation	Children in any African country
**I**ntervention	Human Vaccines for children
**C**omparison	Standard care, do nothing
**O**utcomes	• Cost • Incremental Cost Effectiveness Ratio (ICER) • Cost effectiveness decision • Budget Impact
**T**imeframe	2000 to July 2025

Below are the key words that were used in the search (Table 2).

**Table 2:** The list of key words and their variations.

Key word	Variations
Children	Newborn, infant, children, child, youth
Vaccine	Vaccination, vaccine, immunization,
Specific vaccines	Human papillomavirus vaccine (HPV) Malaria vaccine Hepatitis B vaccine (HepB) Measles, mumps and rubella (MMR) vaccine Measles vaccine (MEAS) Meningococcal Pneumococcal vaccine (PCV) Tuberculosis vaccine (BCG) Tetanus vaccine (TT) Rotavirus virus vaccine (ROTA) Diphtheria vaccine (D, d^4^) Pertussis vaccine (acP) Mumps (MUMPS) Rubella (RUBE) Varicella (chickenpox)
Cost	Direct cost, indirect cost, medical cost, non-medical cost, nonmedical cost, opportunity cost, budget impact analysis
Economic evaluation	Cost effectiveness analysis, cost utility analysis, cost benefit analysis
Africa	Africa, sub-Saharan Africa, Algeria, Angola, Benin, Botswana, Burkina Faso, Burundi, Cameroon, Cape Verde, Central African Republic, Chad, Comoros, Congo, Cote d’Ivoire, Democratic Republic of Congo, Djibouti, Egypt, Equatorial Guinea, Eritrea, Eswatini (Swaziland), Ethiopia, Gabon, Gambia, Ghana, Guinea, Guinea-Bissau, Kenya, Lesotho, Liberia, Libya, Madagascar, Malawi, Mali, Mauritania, Mauritius, Mayotte, Morocco, Mozambique, Namibia, Niger, Nigeria, Réunion, Rwanda, Saint Helena, São Tomé and Príncipe, Senegal, Seychelles, Sierra Leone, Somalia, South Africa, South Sudan, Sudan, United Republic of Tanzania, Togo, Tunisia, Uganda, Zambia, Zimbabwe

Search strings customized to each of the databases were developed.

### Selection of sources of evidence

The articles found through the search were uploaded to Covidence [25] where screening and full text review was done. The uploaded articles were assessed for similarity and duplicates removed. Screening of titles and abstracts of uploaded articles was done by two independent researchers, EM and JK. This was followed by full text review to assess the articles for inclusion into the study based on the inclusion and exclusion criteria. In the event of any conflicts on whether an article fulfils the inclusion criteria, a third researcher (FM) made the final decision.

### Data charting process

A data extraction template was developed and used for recording relevant data that were extracted from included articles. Tables were created to form a basis for data analysis.

### Data items

Some of the data items that were captured from all included economic evaluation studies are author, year of publication, aim of the study, vaccine under consideration, study design, country where the study was conducted, study population, sample size, type of study (e.g., cost effectiveness analysis, cost utility, costing), model used, comparator, currency and year, costing perspective, administration strategy, costs, other key findings, funding source, and conflict of interest. Data items extracted from economic evaluations other than costing studies include outcome measures, time horizon, cost effectiveness threshold used, ICER, cost effectiveness decision. For studies that focused on the use of economic evidence in decision making, we extracted data on author, year of publication, aim of the study, study design, country where the study was conducted, existing structures utilising economic evidence for decision making, challenges and enablers of economic evidence use for decision making among others. Existing structures using economic evidence were identified as such if a study explicitly reports it for example stating whether NITAG or an HTA agency used economic evidence.

### Synthesis of results

We conducted a narrative synthesis of the included studies, structured around key themes such as country, disease or agent targeted, type of economic evaluation, setting, target population, use of economic evidence and policy relevance. The extracted data was analysed descriptively in form of tables, graphs, and maps. The summary measures used in this study were descriptive statistics such as frequencies. Synthesis of the costs and effects of the vaccines was performed for example computing the proportion of the ICER as a proportion of the GDP per capita of the respective country at the time the study was conducted.

### Patient and public involvement

No patient nor the public were involved in this study, as we only reviewed secondary publicly accessible data.

### Ethics and dissemination

This study reviewed secondary data; therefore, it was exempted from application for ethical review.

## Findings

### Studies included in this review

The search of the electronic databases yielded a total of 4037 articles after removal of duplicates. During the title and abstract screening, 3821 articles were excluded at this stage because they did not meet the predefined inclusion criteria. The main reasons for exclusion included studies involving adult populations, which were outside the scope of this review focusing on children; inappropriate study designs; irrelevant interventions; studies conducted outside the African context, as the review was limited to studies from African countries; methodological studies; COVID-19 related studies; and influenza related studies. Following the full-text retrieval and assessment, a total of 216 articles were sought for retrieval, of these 7 articles could not be retrieved despite intensive attempts to access the full-text versions and were therefore excluded.

Hence, the remaining 209 full-text articles were assessed for eligibility, and 69 articles were excluded based on the following reasons: Has no cost or cost-effectiveness (n = 22), adult population (n = 12), wrong intervention (n = 9), wrong setting (n = 8), wrong study design (n = 7), methodological studies (n = 7), vaccine unspecified (n = 1), retracted article (n = 1), non-English language publication (n = 1), and PhD dissertation containing combined papers (n = 1).

**Fig 1:**
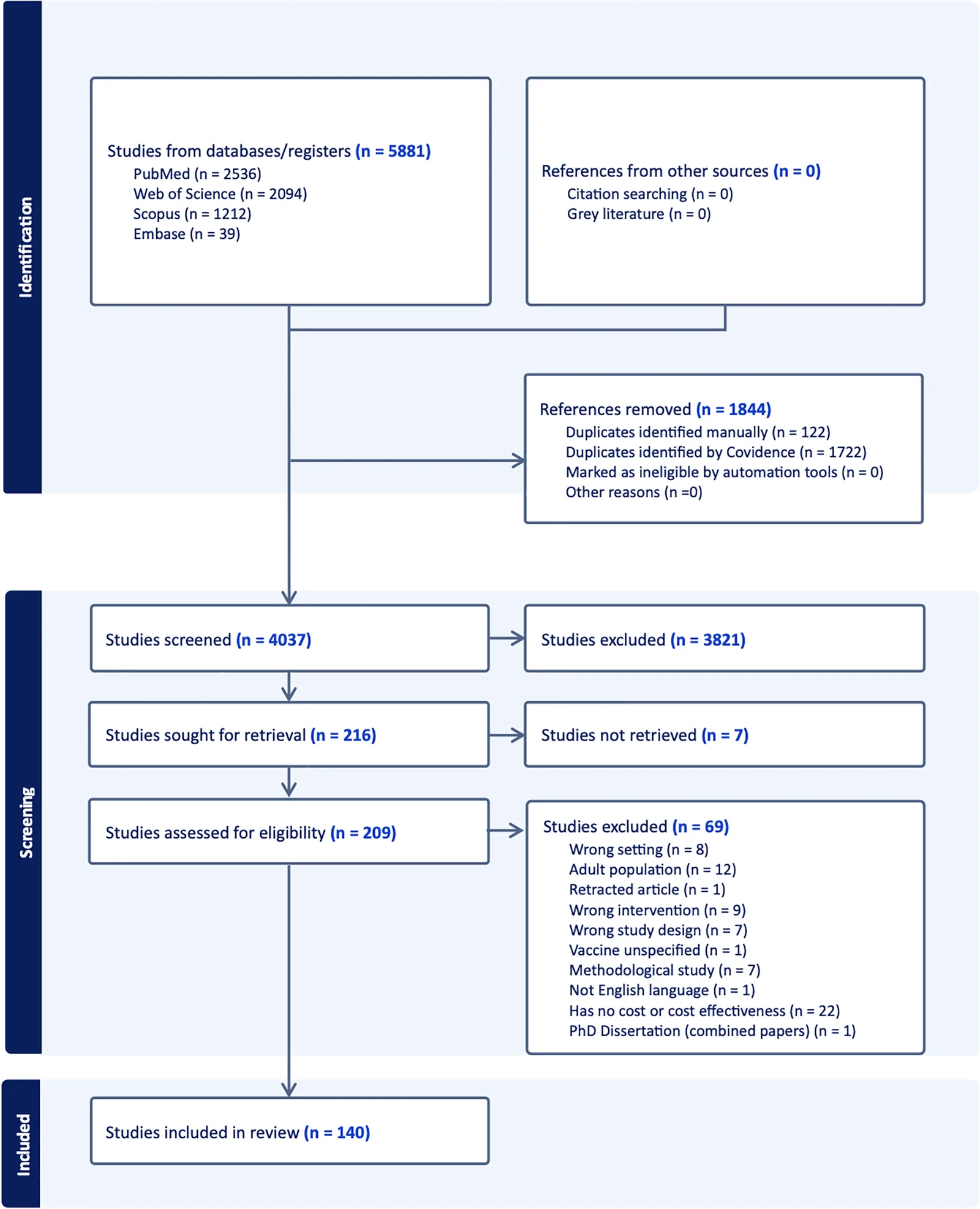
PRISMA chart showing the number of articles assessed for inclusion into the review.

Following the eligibility assessment, a total of 140 articles met the inclusion criteria and were included in the final analysis. Fig 1 presents the PRISMA chart summarizing the study selection process.

### Characteristics of economic evaluation studies included

A total of 129 studies out of the 140 studies included in this review were economic evaluation studies. The remaining eleven studies were non-economic evaluation studies that focused on the use of economic evidence in decision-making in Africa (these are described later in this review). This subsection focuses exclusively on the economic evaluation studies.

**Fig 2:**
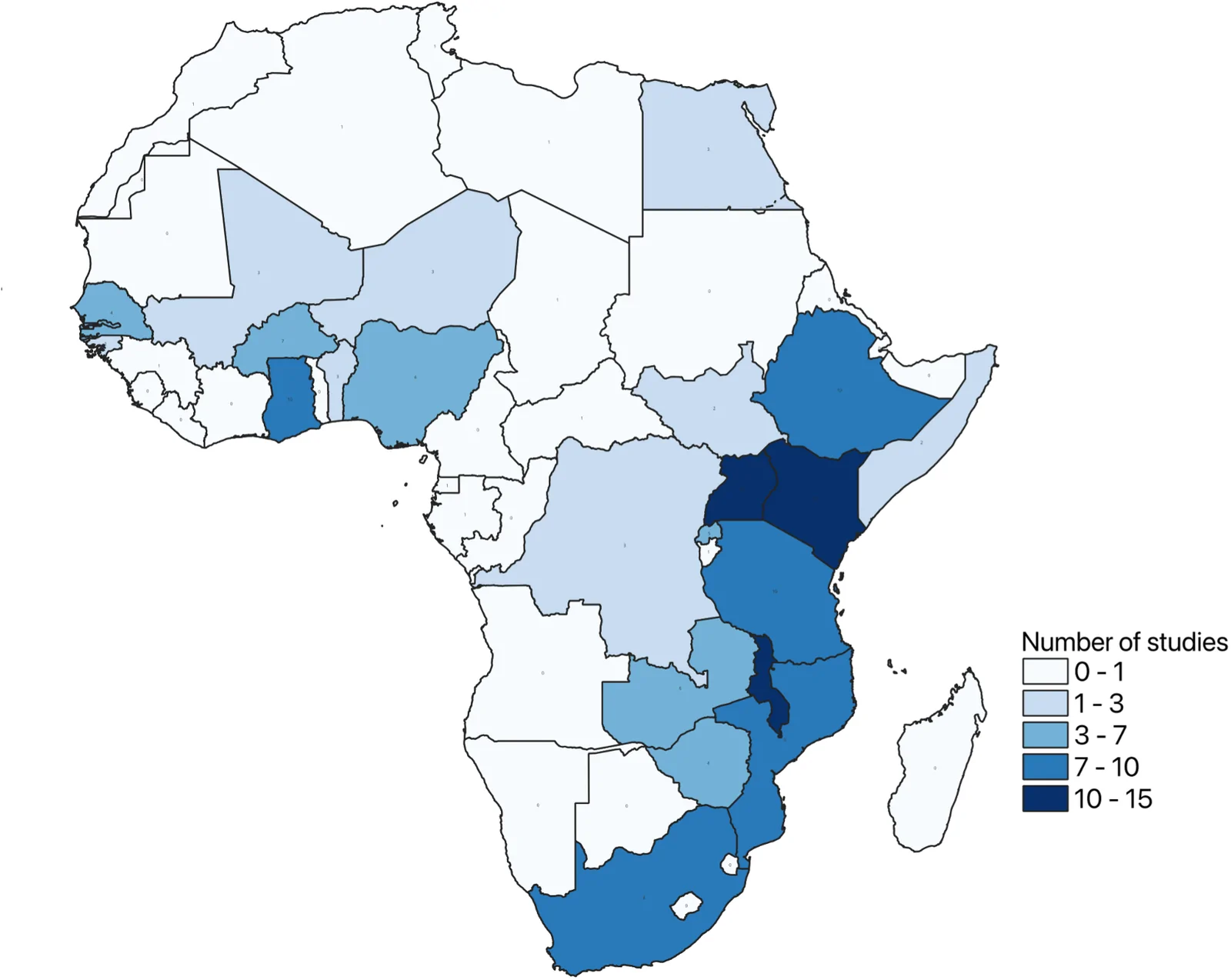
Number of included articles by country. This map was created using map boundaries by Natural Earth (www.naturalearthdata.com) and QGIS software (www.qgis.org/).

The Majority of the studies were conducted in Kenya and Uganda, with each country accounting for over 15 studies, followed by Malawi with 14 studies (Table 3 and Fig 2). The most frequently targeted diseases were HPV, Rota virus, pneumonia, malaria, and measles with each disease being investigated in more than ten studies. The most investigated targeted disease was HPV (n = 34 studies; 26.4%), followed by Rota virus (n = 29 studies; 22.5%), pneumonia (n = 16 studies; 12.4%), malaria (n = 14 studies; 10.9%), and measles (n = 13; 10.1%). Other targeted diseases included hepatitis B (n = 7 studies), Typhoid (n = 5 studies), Meningitis (n = 3 studies), Shigella (n = 3 studies) and Tuberculosis (n = 2 studies).

**Table 3:** Characteristics of included Cost-effectiveness studies.

Article	Country(ies)	Disease/agent targeted	Study type	Setting	Target population	CE threshold decision rule used	Threshold applied to determine CE
Abbott et al 2012 [26]	Ghana	Rota virus	CEA	Sub national	Children <5 years	1-3* GDP per capita	1* GDP per capita
Alkoshi et al 2014 [27]	Libya	Rota virus	CEA	National	Children ≤5 years	1-3* GDP per capita	1* GDP per capita
Anderson et al 2019 [28]	79 low- and lower middle-income countries	ETEC and Shigella disease	CEA	National	Children <5 years	1-3* GDP per capita	3* GDP per capita
Anderson et al 2019 [29]	Democratic Republic of Congo (DRC), Kenya, Zambia, and Zimbabwe	ETEC and Shigella disease	CEA	National	Children <4 years	1-3* GDP per capita	1-3* GDP per capita
Anderson et al 2020 [30]	Nigeria	Rota virus	CEA	National	Children <5 years	–	–
Anderson et al 2023 [31]	102 low-income and middle-income countries	Shigella	CEA	Regional (Africa)	Children <5 years	Comparison with previous CEA decisions	Comparison with previous CEA decisions
Antillón et al 2017 [32]	Kenya	Typhoid	CEA	Sub national	Children 9 months to 25 years	1-3* GDP per capita	1-3* GDP per capita
Arifin et al 2019 [33]	Niger	Meningitis	CEA	National	Children 9 months old	1-3* GDP per capita	3* GDP per capita
Ayieko et al 2013 [34]	Kenya	Pneumonia	CEA	National	Children <5 years	1-3* GDP per capita	3* GDP per capita
Bar-Zeev et al 2016 [35]	Malawi	Rota virus	CEA	National	Children <5 years	1-3* GDP per capita	3* GDP per capita
Bell et al 2020 [36]	Malawi	Malaria	CEA	Sub national	Children 5 –17 months old	–	–
Berry et al 2010 [37]	Malawi	Rota virus	CEA	National	Children <5 years	1-3* GDP per capita	1* GDP per capita
Bishai et al 2011 [38]	Uganda	Measles	CEA	National	Infants 6–11.5 months	–	–
Burger et al 2018 [39]	Uganda	HPV	CEA	National	Girls 9 years old	1-3* GDP per capita	1* GDP per capita
Byberg et al 2017 [40]	Guinea-Bissau	Measles	CEA	National	Children of all ages	–	–
Campos et al 2012 [41]	Kenya, Mozambique, Tanzania, Uganda, and Zimbabwe	HPV	CEA	National	Girls <12 years old	1-3* GDP per capita	3* GDP per capita
Carias et al 2015 [42]	Uganda	Typhoid	CEA	National	Children 5 years old	1-3* GDP per capita	1* GDP per capita
Channing and Sinanovic 2014 [43]	South Africa	Tuberculosis	CEA	National	Children 4 months old	–	–
Debellut et al 2022 [44]	Niger	Rota virus	CEA	National	Children <5 years	0.5* GDP per capita	0.5* GDP per capita
Diop et al 2015 [45]	Senegal	Rota virus	CEA	National	Children <5 years	1-3* GDP per capita	3* GDP per capita
Doshi et al 2015 [46]	Democratic Republic of Congo	Measles	CEA	National	Children 9 months	–	–
Doshi et al 2017 [46]	DRC	Measles	CEA	National	Children <5 years	–	–
Drolet et al 2021 [47]	Uganda, and Nigeria	HPV	CEA	National	Girls 9–12 years old	–	0.5 and 1*GDP per capita
Ekwunife & Lhachimi 2017 [48]	Nigeria	HPV	CEA	National	Girls 9 years old	1-3* GDP per capita	3* GDP per capita
Galactionova et al 2017 [49]	43 African countries	Malaria	CEA	National	Children <18 months	1-3* GDP per capita	3* GDP per capita
Gargano et al 2015 [50]	Somalia	Rota virus	CEA	National	New-borns	1-3* GDP per capita	3* GDP per capita
Gargano et al 2017 [51]	;South Sudan	Haemophilus influenzae type b (Hib) and Pneumonia	CEA	National	Children <2 years	1-3* GDP per capita	1* GDP per capita
Goldie et al 2008 [52]	72 GAVI-eligible countries	HPV	CEA	National	Girls 9–12 years old	1-3* GDP per capita	3* GDP per capita
Gosset et al 2021 [53]	Burkina Faso	Hepatitis B	CEA	National	Children <16 weeks	–	0.5* GDP per capita
Griffiths et al 2005 [54]	Mozambique	Hepatitis B	CEA	National	New-borns	Comparison with previous CEA decisions	Comparison with previous CEA decisions
Griffiths et al 2006 [55]	South Africa	Polio	CEA	National	New-borns	–	–
Guimarães et al 2022 [56]	Mozambique	Rota virus	CEA	National	Children <5 years	0.5* GDP per capita	0.5* GDP per capita
Guimarães et al 2023 [57]	Mozambique	HPV	CEA	National	Girls 9–14 years old	1* GDP per capita	35%* GDP per capita
Haidari et al 2017 [58]	Mozambique	HPV	Costs and effects	National	Girls 10 years old	–	–
Ibrahim et al 2024 [59]	Ghana	Pneumonia	CEA	National	Children <5 years	1-3* GDP per capita	3* GDP per capita
Janko et al 2022 [60]	195 countries	Rota virus	CEA	National	Children under 5 years	1-3* GDP per capita	various
Jit et al 2014 [61]	179 countries	HPV	CEA	National	Girls 12 years old	1-3* GDP per capita	3* GDP per capita
Kaucley & Levy 2015 [62]	Benin	Measles	CEA	Sub national (Rural)	Children <5 years	Comparison with previous CEA decisions	Comparison with previous CEA decisions
Kebede et al 2019 [63]	Ethiopia	Pneumonia	CEA	National	Children 2–39 months	1-3* GDP per capita	3* GDP per capita
Khiari et al 2024 [64]	Tunisia	HPV	CEA	National	Girls 10 years old	1-3* GDP per capita	Both 1 and 3* GDP per capita
Kiatpongsan & Kim 2014 [65]	Kenya and Uganda	HPV	CEA	National	Girls 9 years old	1-3* GDP per capita	Both 1 and 3* GDP per capita
Kiendrebeogo et al 2023 [66]	Burkina Faso	HPV	CEA	National	Girls 9 year old	0-1* GDP per capita	1* GDP per capita
Kim et al 2007 [67]	Gambia	Hepatitis B	CEA	National	Infants	Multiple (comparison with previous decisions and 1–3*GDP per capita)	1* GDP per capita
Kim et al 2010 [68]	Gambia	Pneumonia	CEA	National	Children <5 years	1-3* GDP per capita	3* GDP per capita
Kim et al 2011 [69]	72 GAVI-eligible countries	Rota virus and HPV	CEA	National	Rotavirus vaccine: Infants HPV vaccine: girls 9 years old	1-3* GDP per capita	3* GDP per capita
Kuznik et al 2017 [70]	26 countries of the African meningitis belt	Meningitis	CEA	National	Children 1–10 years old	1-3* GDP per capita	3* GDP per capita
Laufer et al 2023 [71]	Mali	Respiratory syncytial virus (RSV)	CEA	National	Infants <36 months	–	–
Lee et al 2019 [72]	Kenya	Measles	CEA	National	Children <24 months of age in geographically hard-to-reach	1-3* GDP per capita	3* GDP per capita
Levin et al 2007 [73]	Ghana	Four thermostable vaccines	CEA	National	Children <5 years	1-3* GDP per capita	3* GDP per capita
Li et al 2015 [74]	South Africa	HPV	CEA	National	Girls 12 years old	1-3* GDP per capita	3* GDP per capita
Memirie et al 2020 [75]	Ethiopia	Hepatitis B	CEA	National	New-borns	1-3* GDP per capita	0.31* GDP per capita
Michaeli et al 2022 [76]	South Africa	HPV	CEA	National	Girls 9–14 year old	–	–
Mohy et al 2024 [77]	Morocco	Rota virus	CEA	National	Children <5 years	1-3* GDP per capita	3* GDP per capita
Moodley et al 2016 [78]	South Africa	HIV	CEA	National	Children 9 years old	–	–
Mwenda et al 2023 [79]	Kenya	HPV	CEA	National	Girls 10–14 year old	0.5* GDP per capita	0.5* GDP per capita
Ndeketa 2020 [80]	Malawi	Malaria	CEA	National	Children 2–10 years	1-3* GDP per capita	3* GDP per capita
Niyibitegeka et al 2021 [81]	Burundi	Rota virus	CEA	National	Children <5 years	1-3* GDP per capita	3* GDP per capita
Nonvignon et al 2018 [82]	Ghana	Rota virus	CEA	National	Children <5 years	1* GDP per capita	1* GDP per capita
Ojal et al 2019 [83]	Kenya	Pneumonia	CEA	National	Children <5 years	1-3* GDP per capita	1* GDP per capita
Ortega et al 2009 [84]	Egypt	Rota virus	CEA	National	Children <5 years	–	–
Patterson 2024 [85]	South Africa	Hepatitis A	CEA	National	Children 9 years	–	–
Pecenka et al 2018 [86]	Ghana and Malawi	Rota virus	CEA	National	New-borns	GNI per capita	GNI per capita
Pecenka et al 2021 [87]	Gambia	Pneumonia	CEA	National	Children <5 years	–	15% per capita GNI
Penny et al 2016 [88]	Ethiopia	Malaria	CEA	Regional (Africa)	Children 5–17 months	–	–
Phillips et al 2023 [89]	Malawi	Typhoid	CEA	National	Children 9 months and 15 year old	–	–
Pugh et al 2019 [90]	Tunisia and Algeria	Pneumonia	CEA	National	Children <1 year	1-3* GDP per capita	1* GDP per capita
Reardon et al 2019 [91]	African region	Hepatitis B	CEA	National	New-borns of HBV-infected mothers in refugee population	–	1* GDP per capita
Ruhago et al 2015 [92]	Tanzania	Rota virus	CEA	National	Children <5 years	1-3* GDP per capita	1* GDP per capita
Sauboin et al 2019 [93]	41 SSA countries	Malaria	CEA	Regional (sub Saharan Africa)	Infants and children	1-3* GDP per capita	3* GDP per capita
Schmit et al 2024 [94]	Burkina Faso	Malaria	CEA	National	Children 5 –17 months old	–	–
Scott et al 2018 [95]	Global	Hepatitis B	CEA	Regional	New-borns	–	–
Seo et al 2014 [96]	Malawi	Malaria	CEA	National	Children <1 year	1-3* GDP per capita	3* GDP per capita
Sevilla et al 2022 [97]	Egypt	Pneumonia	CEA	National	Children <1 year	1-3* GDP per capita	1* GDP per capita
Sibak et al 2015 [98]	Egypt	Pneumonia	CEA	National	Children <5 years	1-3* GDP per capita	3* GDP per capita
Sigei et al 2015 [99]	Kenya and Uganda	Rota virus	CEA	National	Children <5 years	1-3* GDP per capita	1* GDP per capita
Sinanovic et al 2009 [100]	South Africa	HPV	CEA	National	Girls 12 years old	1-3* GDP per capita	3* GDP per capita
Sinha et al 2007 [101]	72 countries	Pneumonia	CEA	National	Children <14 weeks	1-3* GDP per capita	Various
Tate et al 2009 [102]	Kenya	Rota virus	CEA	National	Children <5 years	1-3* GDP per capita	3* GDP per capita
Tate et al 2011 [103]	Uganda	Rota virus and Pneumonia	CEA	National	Children <5 years	1-3* GDP per capita	1-3* GDP per capita
Topazian et al 2023 [104]	Sub Saharan Africa	Malaria	CEA	Regional (Sub Saharan Africa)	Children <5 years	–	–
Touray et al 2011 [105]	Gambia	Pneumonia	CEA	National	Infants 6–14 weeks	1-3* GDP per capita	3* GDP per capita
Tracy et al 2014 [106]	Mali	HPV	CEA	National	Girls 10–14 years old	1-3* GDP per capita	3* GDP per capita
Tseng et al 2011 [107]	Zambia	Tuberculosis	CEA	National	Children <10 years	–	–
Van Hoek et al 2012 [108]	Kenya	Rota virus	CEA	National	Children <5 years	1-3* GDP per capita	3* GDP per capita
Verguet et al 2013 [109]	Ethiopia	Rota virus	CEA	National	Children <5 years	–	–
Vodicka et al 2022 [110]	Ghana	HPV	CEA & BIA	National	Girls 9 year old	4- 40% of GDP per capita	up to 40% of GDP per capita
Wedlock et al 2019 [111]	Benin, Mozambique and Niger	Measles	CEA	National	Children 5 years old	1-3* GDP per capita	3* GDP per capita
Wenger 2024 [112]	Malawi	Rota virus	CEA	National	Infants	–	0.5* GDP per capita
Wenger et al 2025 [113]	Malawi	Rota virus	CEA	National	Children <5 years	0.5* GDP per capita	0.5* GDP per capita
Wondimu et al 2022 [114]	Ethiopia	HPV	CEA	National	Girls 12 year old	1-3* GDP per capita	1* GDP per capita
Zimmermann et al 2019 [115]	Nigeria	Measles	CEA	National	Children <10 years	–	–

SSA: Sub Saharan Countries, HPV: Human papilloma virus, CEA: Cost effectiveness analysis, BIA: Budget impact analysis, GDP: Gross domestic product.

Most studies were cost effectiveness studies (n = 90 studies; 69.8%), followed by costing studies (n = 33 studies; 25.6%). Other studies included budget impact analysis, cost benefit analysis and cost and effects estimations. The majority of studies provided national level estimates (117, 90.7%) while sub national and regional studies accounted for each six studies each. Regarding study populations, the most commonly reported study population were children under five years (n = 38 studies; 29.5%) followed by newborns (n = 8 studies; 6.2%), girls aged nine years (n = 7 studies; 5.4%), and infants (n = 6 studies; 4.7%).

In addition, most studies did not mention report who commissioned the study. Only seven studies provided those this information, of which six were commissioned by either or both National Governments (through Ministries of Health) and WHO [47,88,98,104,116,117], while one study was commissioned by the Gates Foundation [32].

Studies used different cost-effectiveness decision rules to determine cost-effectiveness of vaccine interventions, with 1–3 times GDP per capita being the most commonly used decision rule. Other decision rules included 0.5 times GDP per capita, 1 GDP per capita, and comparisons with previous CEA studies. The columns “CE threshold decision used” and “Threshold applied to determine CE” were included to differentiate the decision rule or framework applied from the specific numerical threshold used, thereby enhancing transparency and allowing comparison of how different threshold choices influence cost-effectiveness conclusions. The detailed study characteristics are available in Table 3–5.

**Table 4:** Characteristics of included Costing studies.

Article	Country(ies)	Disease/agent targeted	Study type	Setting	Target population
Alonso et al 2019 [118]	Mozambique	HPV	Costing	Sub national (Rural)	Girls 10 years old
Baral et al 2021 [119]	Kenya and Malawi	Malaria	Costing	National	Infants
Baral et al 2023 [117]	Ghana, Kenya, and Malawi	Malaria	Costing	National	Children <2 years
Brennan et al 2022 [120]	Senegal	HPV	Costing	National	Girls 9 years old
Byberg et al 2017 [121]	Guinea-Bissau	Measles	Costing	Sub national (Rural)	Children <2 years
Colombini et al 2015 [122]	Burkina Faso	Meningitis	Costing	National	Children 1–29 years
Dayan et al 2004 [123]	Zambia	Measles	Costing	National	New-borns
Debellut et al 2022 [124]	Malawi	Typhoid	Costing	National	Children 9 months - 15 years
Debellut et al 2024 [125]	Malawi	Typhoid	Costing	National	Children 9 months -14 years
Diawara et al 2023 [126]	Mali and Burkina Faso	Malaria	Costing	National	Children <5 years
Galactionovan et al 2015 [127]	Burkina Faso, Ghana, Kenya, Senegal, Tanzania, and Uganda	Malaria	Costing	National	Infants at 6–12 weeks; children at 6–9 months
Griffiths et al 2009 [128]	Ethiopia	Hepatitis B	Costing	National	Children
Griffiths et al 2016 [129]	Zambia	Measles, Pneumonia, Rota virus	Costing	National	Infants
Hidle et al 2018 [130]	Zimbabwe	HPV	Costing	National	Girls 10 years old
Hidle et al 2022 [131]	Zimbabwe	HPV	Costing	National	Girls 10–14 years old
Hsiao et al 2023 [132]	Tanzania	HPV	Costing	National	Girls aged 9 –14 years old
Hutubessy et al 2012 [116]	Tanzania	HPV	Costing	National	Girls 9–13 years old
Ilboudo et al 2022 [133]	Benin	Pneumonia	Costing	National	Infants
Levin et al 2013 [134]	Uganda	HPV	Costing	National	Children <5 years
Levin et al 2014 [135]	Uganda, and Tanzania	HPV	Costing	National	Girls 9–13 years old
Madsen et al 2014 [136]	Malawi	Rota virus	Costing	National	Children <5 years
Mak et al 2024 [137]	Zambia	Measles	Costing	National	Children <5 years
Mvundura 2024 [138]	Ethiopia	HPV	Costing	National	Girls 14 years old
Mvundura et al 2023 [139]	Ethiopia, Rwanda, Senegal, and Uganda	HPV	Costing	National	Girls
Ngabo et al 2015 [140]	Rwanda	Pneumonia, Rota virus and HPV	Costing	National	HPV vaccine: Girls 9–13 years Pneumococcal and rotavirus vaccines: Children <12 months
Quentin et al 2012 [141]	Tanzania	HPV	Costing	Regional	Girls school going
Sicuri et al 2019 [142]	Burkina Faso, Ghana, Kenya, Mozambique, and Tanzania	Malaria	Costing	National	Children <5 years
Simuyemba et al 2024 [143]	Zambia	HPV	Costing	National	Girls 14 years old
Slavkovsky et al 2024 [144]	Ethiopia, Rwanda, and Uganda	HPV	Costing	National	Adolescents
Soi et al 2019 [145]	Mozambique	HPV	Costing	National	Girls 10 years old
Usuf et al 2014 [146]	Gambia	Pneumonia	Costing	National	New-borns
**Article**	**Country(ies)**	**Disease/agent targeted**	**Study type**	**Setting**	**Target population**
Vaughan et al 2020 [147]	Tanzania	Numerous	Costing	National	Children <18 months

HPV: Human papilloma virus.

**Table 5:** Characteristics of included Cost Benefit Analysis and Budget Impact Analysis studies.

Article	Country(ies)	Disease/agent targeted	Study type	Setting	Target population
Jeuland et al 2009 [148]	Mozambique	Cholera	CBA	National	Children 1–4 years
Kananura et al 2024 [149]	Uganda	Rota virus	CBA	National	Children <5 years
Levin et al 2011 [150]	Ethiopia, and Uganda	Measles	CBA	National	Children ≤16 years
Mohy et al 2023 [151]	South Africa	Rota virus	CBA	National	Children <5 years
Ndikumukiza 2021 [152]	Rwanda	Malaria	BIA	National	Children <5 years
Okafor & Ekwunife 2021 [153]	Central African Republic, Chad, Comoros, Equatorial Guinea, Gabon, Guinea, Somalia, and South Sudan	Rota virus	CBA	National	Children <1 year

BIA: Budget impact analysis, CBA: Cost benefit analysis, GDP: Gross domestic product.

Table 5 describes the 7 studies that analyzed vaccine interventions using economic evaluations other than cost-effectiveness analysis or. Of these, five studies were CBAs and one study each applied a BIA and a cost and effects approach.

### Number of publications of included economic evaluation studies from 2000 to 2025

**Fig 3:**
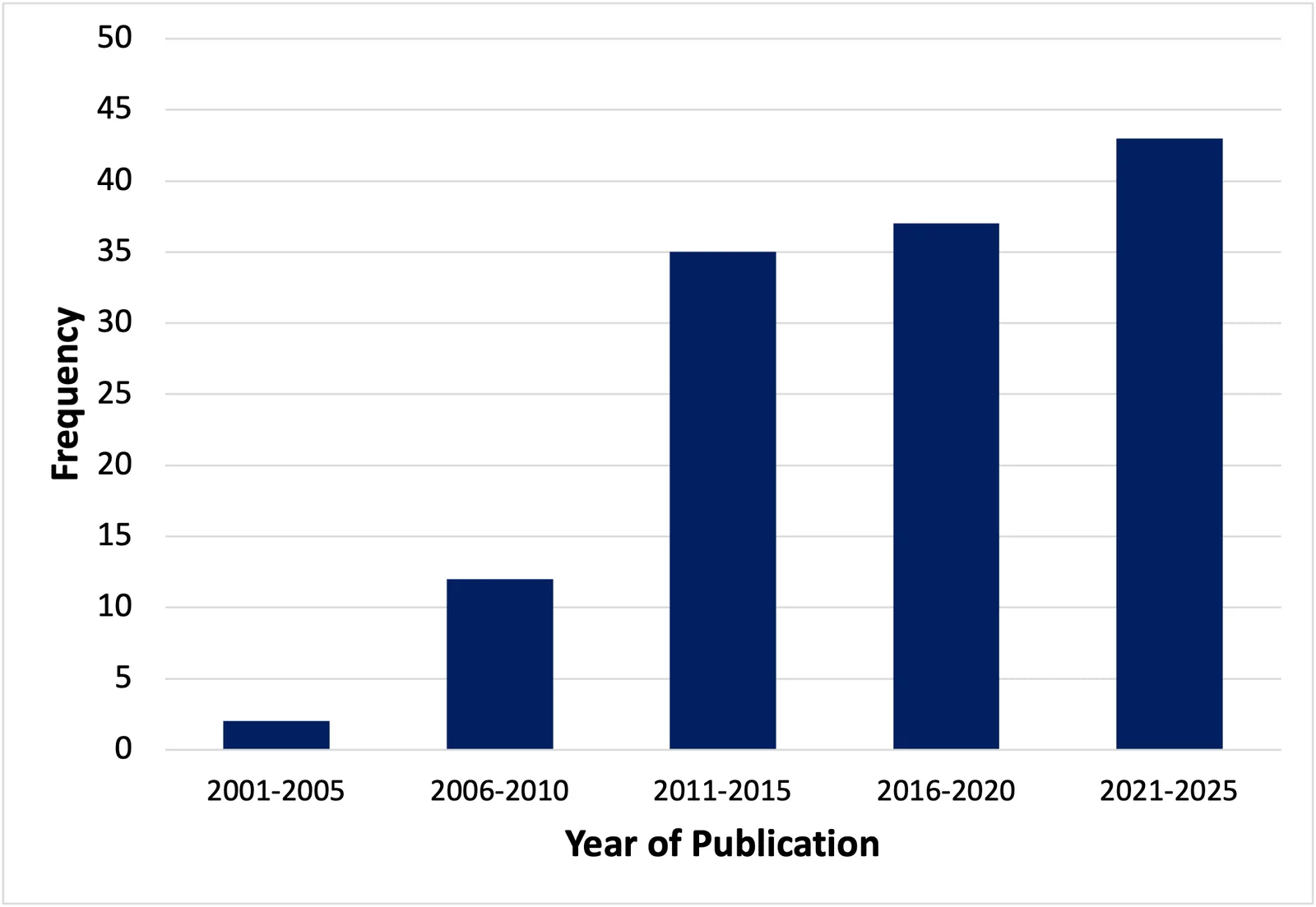
Graph showing the number of economic evaluation studies by year of publication.

There has been an increasing trend in the number of studies published since 2000. The 2000–2005 period had the least number of published articles (n = 2 studies) while 2021–2025 period has the greatest number of included articles (n = 43 studies). Below is Fig 3 showing the frequency of published articles by period.

### Cost effectiveness studies for child vaccines

The details of the cost effectiveness studies are presented in S1 Table. Of the 91 cost effectiveness studies included in the review, 24 studies investigated the cost effectiveness of Rota virus vaccines followed by HPV vaccines (n = 18 studies), pneumococcal vaccines (n = 11 studies), malaria vaccines (n = 7 studies) and hepatitis B vaccine (n = 7 studies). Other vaccines investigated include Shigella vaccine, measles vaccine, HIV vaccine, Hepatitis A vaccine, BCG, PCV among others. Majority of the studies compared the intervention to no vaccine (n = 59 studies; 65.6%) while only 7 studies (7.8%) compared the intervention with the status quo or standard care. Most studies used the societal perspective (n = 26 studies), followed by government perspective (n = 17 studies), and provider perspective (n = 13 studies). Secondary data and or literature was the main source of data in 79 of the 91 cost effectiveness studies (87.8%). The rest of the studies were based on primary data. Most reported health outcome measure was DALYs averted (n = 70 studies; 77.8%). Only 12 studies (13.3%) reported health outcomes using QALYs gained. Other health outcome measures that were reported by studies include deaths averted, cases averted, and life years gained. Most studies reported costs in United States dollars (n = 79 studies; 87.8%). 1–3 times GDP per capita was the commonest cost effectiveness decision rule (n = 51 studies; 58.0%) used to determine whether an intervention is cost effective or not. Of the studies that used 1–3 times GDP per capita as the decision rule, studies that used 1X GDP per capita as the threshold were 32 in number, followed by those that used 3X GDP per capita (n = 15 studies) while two studies used both thresholds.

### Author affiliation and funding for included economic evaluation studies

**Fig 4:**
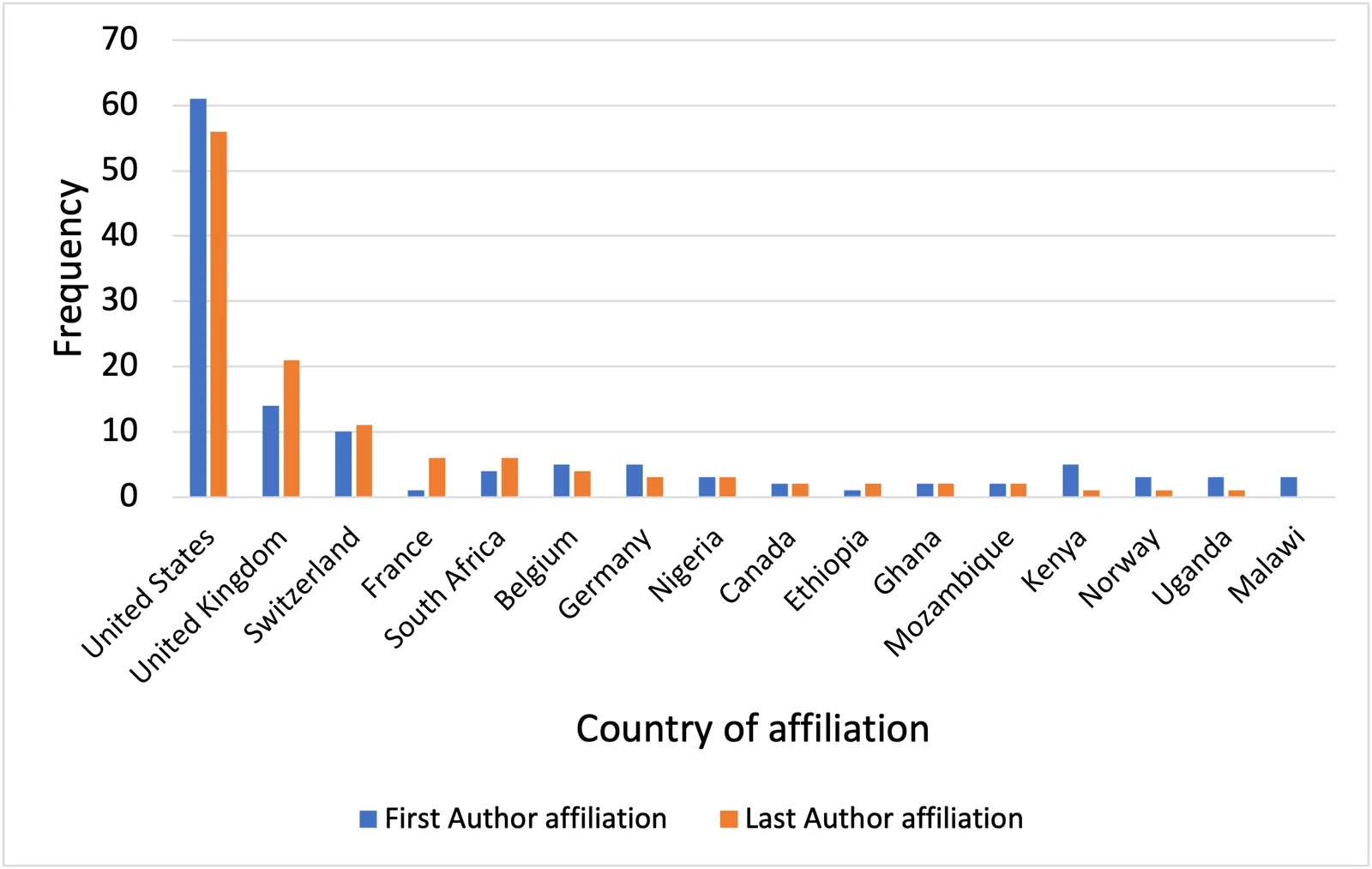
Graph showing the frequency of country of affiliation of first and last author of included studies.

The first and last authors were majorly affiliated to institutions based in countries outside the African continent. Majority of the first authors were based in United States of America (n = 61; 46.5%) followed by United Kingdom (n = 14; 10.9%) and Switzerland (n = 10; 7.8%). The affiliation of the last authors (mostly senior authors) follows a similar trend as the first authors where majorly based in United States of America n = 56 (43.4%), followed by United Kingdom (n = 21; 16.3%) and Switzerland (n = 11; 8.5%). The graph below (Fig 4) shows the countries with the most frequencies for first and last author affiliation.

**Fig 5:**
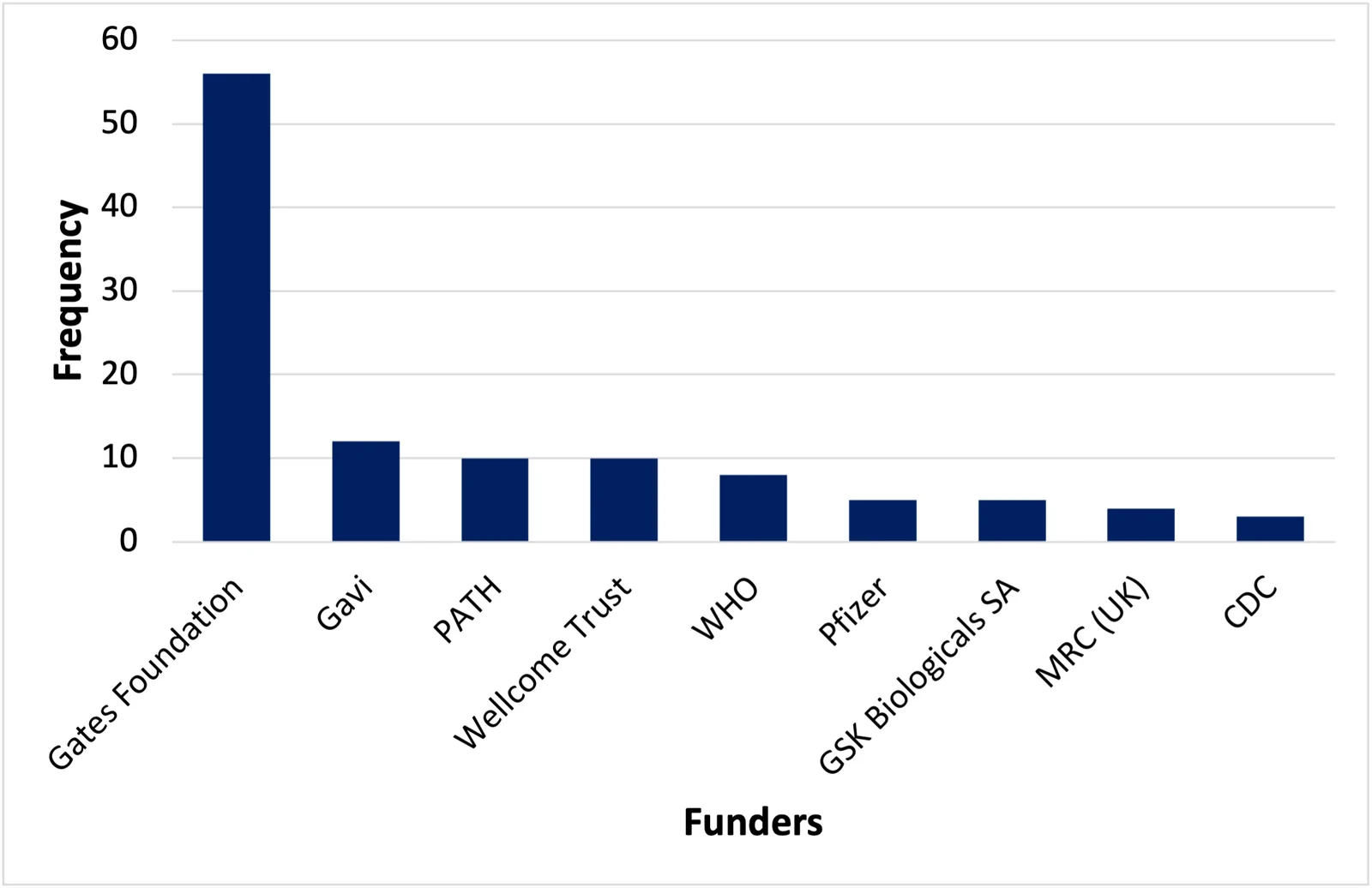
Names of funders that financed more than two of the included articles.

Funders that financed more than two studies included Gates Foundation (formerly known as the Bill and Melinda Gates foundation), Gavi, PATH, Wellcome Trust, WHO, Pfizer, GlaxoSmithKline Biologicals SA (GSK Biologicals SA), Medical Research Council of United Kingdom (MRC UK) and Centres for Disease Control and Prevention United States of America (CDC). Gates Foundation funded the greatest number of studies (n = 56 studies) equivalent to 43.4% of all economic evaluation studies included in this review. This was followed by Gavi (n = 12 studies; 9.3%), PATH (n = 10 studies; 7.8%), Wellcome Trust (n = 10 studies; 7.8%) and WHO (n = 8 studies; 6.2%). Fig 5 shows frequency of economic evaluation studies financed by the most frequent funders. S2 Table is a table Showing the affiliation of the first and last authors and the respective study funders.

### The magnitude of the ICER of vaccine interventions in relation to the respective GDP per capita of the country

The cost-effectiveness of vaccine interventions represented by the ICER, relative to GDP per capita, varied across diseases, vaccine types, and perspectives. For rotavirus vaccines, most evaluations reported ICERs below 0.5 times GDP per capita from the government perspective. Specifically, Rotarix and the generic Rota virus vaccine had 79% and 75% of evaluations respectively with ICERs below 0.5 times the GDP per capita, while Rotasill, RotaTeq and Rotavac, had less than 50% or less of the evaluations with ICERs below 0.5 times GDP per capita. When analyses included Gavi support under the government perspective, all evaluations reported ICERs below 0.5 times GDP per capita. Similarly, ICERs tended to be lower when the societal perspective was applied.

For typhoid vaccines, only three evaluations were identified, all reporting ICERs between 0.5 and 1.0 GDP per capita under the government and health systems perspectives, indicating less favorable results compared to rotavirus vaccines. Similarly, the single RSV vaccine study reported an ICER between 0.5 and 1.0 GDP per capita from a government perspective.

Pneumococcal conjugate vaccines (PCV) demonstrated mixed results. PCV13 generally had ICERs below 0.5 GDP per capita under the government, payer and societal perspectives, while older formulations (PCV7 and PCV9) showed less favorable ICERs. Measles and malaria vaccines had consistently low ICERs below 0.5 GDP per capita across most perspectives.

HPV vaccines, including Cecolin, Cervarix, and Gardasil variants, generally had ICERs below 0.5 GDP per capita from all the perspectives. Hepatitis B vaccines also showed favorable cost-effectiveness estimates, with all evaluations reporting ICERs below 0.5 GDP per capita. In contrast, the single hepatitis A vaccine study reported an ICER above 3.0 GDP per capita, indicating low value for money.

Overall, vaccines consistently exhibit a good value for money. The value for money increases to the countries when Gavi support is considered. Vaccines for rotavirus, measles, malaria, HPV, and hepatitis B consistently demonstrated high economic value, particularly when Gavi support was considered. Typhoid and RSV vaccines had moderate value for money, while hepatitis A vaccination appeared least favorable. With Gavi support included in the economic evaluations, the ICERs from a government perspective became lower. The ICER estimates from societal perspective were more likely to be lower compared to estimates from other perspectives (Table 6).

**Table 6:** The proportion of studies categorized by the ICER of vaccine interventions as a percentage of the GDP per capita in each cost effectiveness perspective.

Disease	Vaccine	Number of evaluations	ICER as a proportion of the GDP																			
			Government perspective n(%)				Government perspective with Gavi support n(%)				Payer perspective n(%)				Health system perspective n(%)				Societal perspective n(%)			
			<0.5	0.5- < 1.0	1.0- < 3.0	>3.0	<0.5	0.5- < 1.0	1.0- < 3.0	>3.0	<0.5	0.5- < 1.0	1.0- < 3.0	>3.0	<0.50	0.5- < 1.0	1.0- < 3.0	>3.0	<0.5	0.5- < 1.0	1.0- < 3.0	>3.00
Rota virus	Rotarix	20	11 (79%)	3 (21%)	0 (0%)	0 (0%)	4 (100%)	0 (0%)	0 (0%)	0 (0%)	–	–	–	–	–	–	–	–	7 (100%)	0 (0%)	0 (0%)	0 (0%)
	Rotasill	9	2 (33%)	2 (33%)	1 (17%)	1 (17%)	3 (100%)	0 (0%)	0 (0%)	0 (0%)	–	–	–	–	–	–	–	–	3 (100%)	0 (0%)	0 (0%)	0 (0%)
	RotaTeq	4	1 (50%)	0 (0%)	1 (50%)	0 (0%)	–	–	–	–	–	–	–	–	–	–	–	–	2 (67%)	0 (0%)	1 (33%)	0 (0%)
	Rotavac	8	2 (50%)	2 (50%)	0 (0%)	0 (0%)	3 (100%)	0 (0%)	0 (0%)	0 (0%)	–	–	–	–	–	–	–	–	2 (100%)	0 (0%)	0 (0%)	0 (0%)
	Rotavirus vaccine	11	3 (75%)	1 (25%)	0 (0%)	0 (0%)	–	–	–	–	0 (0%)	1(33%)	0 (0%)	2 (67%)	2 (100%)	0 (0%)	0 (0%)	0 (0%)	6 (75%)	1 (13%)	0 (0%)	1 (13%)
Typhoid	Typhoid conjugate vaccine (TCV)	2	0 (0%)	1 (100%)	0 (0%)	0 (0%)	–	–	–	–	–	–	–	–	0 (0%)	1 (100%)	0 (0%)	0 (0%)	–	–	–	–
	Vi capsular polysaccharide (ViCPS) vaccine	1	–	–	–	–	–	–	–	–	–	–	–	–	0 (0%)	1 (100%)	0 (0%)	0 (0%)	–	–	–	–
Respiratory syncytial virus (RSV)	RSV vaccine	1	0 (0%)	1 (100%)	0 (0%)	0 (0%)	–	–	–	–	–	–	–	–	–	–	–	–	–	–	–	–
Pneumonia	PCV vaccination	3	1 (100%)	0 (0%)	0 (0%)	0 (0%)	–	–	–	–	–	–	–	–	1 (100%)	0 (0%)	0 (0%)	0 (0%)	2 (100%)	0 (0%)	0 (0%)	0 (0%)
	PCV 10	3	–	–	–	–	–	–	–	–	0 (0%)	1 (100%)	0 (0%)	0 (0%)	–	–	–	–	1 (50%)	0 (0%)	1 (50%)	0 (0%)
	PCV 13	7	3 (75%)	0 (0%)	1 (25%)	0 (0%)	–	–	–	–	2 (100%)	0 (0%)	0 (0%)	0 (0%)	–	–	–	–	3 (75%)	0 (0%)	1 (25%)	0 (0%)
	PCV 7	1	0 (0%)	0 (0%)	1 (100%)	0 (0%)	–	–	–	–	–	–	–	–	–	–	–	–	–	–	–	–
	PCV 9	1	–	–	–	–	–	–	–	–	–	–	–	–	–	–	–	–	0 (0%)	1 (100%)	0 (0%)	0 (0%)
Measles	MCV	8	2 (100%)	0 (0%)	0 (0%)	0 (0%)	–	–	–	–	–	–	–	–	0 (0%)	2 (67%)	1 (33%)	0 (0.0%)	2 (100%)	0 (0%)	0 (0%)	0 (0%)
Malaria	RTS,S	3	1 (100%)	0 (0%)	0 (0%)	0 (0%)	–	–	–	–	–	–	–	–	1 (100%)	0 (0%)	0 (0%)	0 (0.0%)	2 (100%)	0 (0%)	0 (0%)	0 (0%)
HPV	Cecolin	4	2 (100%)	0 (0%)	0 (0%)	0 (0%)	1 (100%)	0 (0%)	0 (0%)	0 (0%)	–	–	–	–	–	–	–	–	2 (100%)	0 (0%)	0 (0%)	0 (0%)
	Cervarix	8	4 (100%)	0 (0%)	0 (0%)	0 (0%)	1 (100%)	0 (0%)	0 (0%)	0 (0%)	–	–	–	–	1 (100%)	0 (0%)	0 (0%)	0 (0.0%)	4 (100%)	0 (0%)	0 (0%)	0 (0%)
	Gardasil-4	4	2 (100%)	0 (0%)	0 (0%)	0 (0%)	1 (100%)	0 (0%)	0 (0%)	0 (0%)	–	–	–	–	–	–	–	–	–	2 (100%)	0 (0%)	0 (0%)
	Gardasil-9	5	1 (100%)	0 (0%)	0 (0%)	0 (0%)	–	–	–	–	–	–	–	–	2 (67%)	1 (33%)	0 (0%)	0 (0.0%)	1 (50%)	0 (0%)	1 (50%)	0 (0%)
	HPV vaccine	5	0 (0%)	0 (0%)	1 (100%)	0 (0%)	–	–	–	–	0 (0%)	1 (100%)	0 (0%)	0 (0%)	–	–	–	–	3 (100%)	0 (0%)	0 (0%)	0 (0%)
HIV	HIV vaccine	1	1 (100%)	0 (0%)	0 (0%)	0 (0%)	–	–	–	–	–	–	–	–	–	–	–	–	–	–	–	–
Hepatitis B	HBV vaccine	3	1 (100%)	0 (0%)	0 (0%)	0 (0%)	–	–	–	–	1 (100%)	0 (0%)	0 (0%)	0 (0%)	–	–	–	–	2 (100%)	0 (0%)	0 (0%)	0 (0.0%)
	HepB-BD + HepB3	2	–	–	–	–	–	–	–	–	–	–	–	–	1 (100%)	0 (0%)	0 (0%)	0 (0%)	1 (100%)	0 (0%)	0 (0%)	0 (0.0%)
Hepatitis A	hepatitis A vaccination	1	0 (0%)	0 (0%)	0 (0%)	1 (100%)	–	–	–	–	–	–	–	–	–	–	–	–	–	–	–	–

CE– Cost effectiveness.

### Economic evidence use in decision making in Africa

Eleven qualitative studies reported on the use of economic evidence in health sector decision making across African countries, including Ethiopia (n = 3) [154–156], Tanzania (n = 2) [157,158], Ghana [159], Liberia [160], Kenya [161], Malawi [162], Uganda [163] (n = 1 each), and one multi-country study covering Ethiopia, Kenya, Malawi, Mozambique [164]. Eight studies explored stakeholder perceptions, while three described actual decision-making processes involving economic evidence [154,158,160]. Use of economic evidence was generally limited. The economic evidence used in decision making primarily focused on costing and was used for planning, resource mobilisation, intervention scale up, resource tracking and procurement [155,164]. More comprehensive economic evidence combining costs and health outcomes were typically used in collaboration with external partners in projects like for example updating the essential medicines lists, health benefit packages among others [157,158,160,161,164]. Most countries lacked formal structures to support evidence use, though some, such as Kenya, Ethiopia, and Ghana, were establishing mechanisms like HTA units and technical working groups [159,161,164]. Reported barriers to using economic evidence in decision making include presence of inadequate, fragmented, inaccessible data, limited available capacity to generate and use economic evidence, low levels of awareness of economic evidence, limited political support, over dependence on donor funds that are already earmarked, insufficient coordination of stakeholders, presence of inadequate financial resources to fund generation and use of economic evidence, absence of locally generated economic evidence, cultural biases among others. The identified enablers of economic evidence use were collaborations with external partners, willingness of stakeholders to get involved, and targeted training initiatives.

## Discussion

In the face of ever-escalating resource constraints and declining external financing for health, economic evaluation evidence can play an important role in helping policy makers assess the value, affordability, and sustainability of vaccine programmes. This review identified a growing but still limited body of literature on vaccine economic evaluation and the use of economic evidence in Africa. The findings suggest that the challenge facing vaccine policymaking on the continent is not simply a lack of studies, but weaknesses across the broader economic evidence ecosystem. Although the production of economic evidence has increased over time, it remains concentrated in a small number of countries, heavily reliant on external actors, and only weakly integrated into policy processes. These findings are discussed through the lenses of the supply, demand, and use of economic evidence.

### Supply of economic evidence

Our findings indicate that the supply of economic evidence for vaccine policymaking in Africa remains limited and unevenly distributed. Although the number of studies has increased over time, economic evaluations are concentrated in a small number of countries, particularly Kenya, Uganda, and Malawi. The current economic context of diminishing development assistance for health in Africa [165] should be yielding more enthusiasm for the generation of economic evidence in vaccine policy, yet the field has struggled to define its role within the immunization landscape in Africa.

These findings suggest that the production of economic evidence remains heavily dependent on external technical and financial support, raising concerns about sustainability, local ownership, and alignment with national policy priorities. The limited number of economic evaluation studies likely reflects the limited funding available for research in Africa. Available studies are funded by a handful of funders based in the global north, with limited funding by national governments in Africa. This finding is similar to other studies that have reported on the lack of in-country funding for research on the Africa continent [166–169]. Some authors have reported that the low levels of local funding could be a result of the low value placed on research evidence by policy makers and their skepticism of its use in policy making [167,170]. African governments should promote the utilization of already established tools that can speed up generation of economic evidence at a less cost for example the UNIVAC and COSTVAC tools used for estimation of cost effectiveness and vaccine cost respectively that have been developed by the Pan American Health [171]. To compliment that and for sustainability, the governments should consider allocating resources to research aimed at generating evidence relevant for policy making amidst the existing financial and economic constraints.

The concentration of studies on HPV and rotavirus vaccines likely reflects a combination of disease burden, donor priorities, and policy interest. Both vaccines have received substantial support from Gavi and other global partners over the past two decades and have been the subject of extensive vaccine introduction and sustainability discussions [5]. Consequently, countries and development partners have had strong incentives to generate evidence on their affordability, implementation costs, and value for money.

In addition, authorship of economic evaluation studies is dominated by researchers based in institutions outside of Africa (mostly from the organization for Economic Cooperation and Development (OECD) countries). This finding is not unique as Panzer et al [172] and Hollingworth et al [166] have reported about the same. Authorship could be influenced by the source of funding. Majority of the funding is from the global north and so are most first and senior authors. This implies that local researchers and policy makers generally have a limited role in the formulation of research questions and execution of studies, potentially rendering many studies less relevant to policy and practice [167,170,172]. This exposes the continent to leaving local research priorities unaddressed and undermines the development of health economics capacity in the region. More research is needed to understand the dynamics between funding, authorship and policy making to inform future research funding calls and policy making.

It is clear from the limitations cited in the vast majority of the studies that economic evaluations in Africa take place in data scarce environment. Over-reliance on data from external sources or models rather than country level empirical data is commonplace [18,173]. Very few countries in Africa (Egypt [174], Ethiopia [175], Ghana [176], Morocco [177], Tunisia [178] and Zimbabwe [179] have population-based value sets that are used in calculating health outcome measures, notably QALYs. As with clinical data, authors have raised the general lack of country level- cost data [180]. As such, the literature was characterized by significant heterogeneity in the considered range of ICERs or costs per health outcome reported. The scarcity of context-specific data likely constrained the evaluation of multiple policy alternatives, leading most studies to assess vaccine interventions against a no-intervention comparator. These data quality challenges render many economic evaluation studies less relevant to some of the contextual questions that vaccine policy makers in Africa face [166].

### Demand for economic evidence

The limited volume of economic evaluation studies may not only reflect constraints in evidence production but may also indicate limited demand for economic evidence within vaccine decision-making systems [167,170]. Unlike some high-income settings where economic evaluation is routinely incorporated into reimbursement and technology adoption decisions, few African countries formally require cost-effectiveness or budget impact evidence for vaccine adoption decisions [18,181]. Although NITAGs and HTA initiatives are expanding across the continent, economic evidence remains only one of several inputs into decision making and is often not systematically required [16].

Although economic evaluation is encouraged to be used for vaccine decision making [5,6,17,182], it has not been formally adopted as a requirement for vaccine policy in Africa [16]. Until recently, immunization was a relatively small component of the national budget and funded as a vertical project and largely escaped scrutiny in national planning and budgets. About 48% of vaccine budgets in Africa is funded by donors, notably Gavi, UNICEF, WHO, Gates Foundation, and others, who have provided countries with vaccines, technical support, vaccine introduction grants, etc [183]. A number of countries in Africa do not have an institutional setup that facilitates the application of economic evidence to support economic evidence [169,181].

Further, the policy making process is largely dominated by medical professionals with limited training in economics [184–186]. Recently, Gavi and WHO have encouraged countries to deliberately integrate economic evidence into vaccine policy through the establishment of NITAGs at country level and Gavi has itself used economic evidence in its vaccine investment strategy (VIS) process [182]. However, studies have shown that conducting economic appraisal of evidence is not formally a part of NITAG scope of work [186]. Gavi does not require countries to perform cost-effectiveness analysis. Indeed, experience around the world shows that economic evidence use is institutionalised within broader priority setting initiatives such as HTA [173, 187, 188]. There is a growing need to institutionalise HTA as part of routine policy and priority-setting processes. Strengthening HTA systems could enhance the generation, appraisal, and use of economic evidence, reduce reliance on ad hoc decision-making, and support transparent and sustainable vaccine investment decisions in resource-constrained settings.

### Use of economic evidence in decision-making

Even when economic evidence is available, its use in policy decisions remains constrained. The studies included in this review suggest that economic evidence is most frequently used for planning, budgeting, procurement, and donor engagement rather than for formal priority-setting or vaccine adoption decisions. Countries with emerging HTA structures and stronger technical capacity appeared more likely to use economic evidence systematically. However, barriers including limited technical expertise, fragmented data systems, insufficient institutionalisation of HTA processes, and dependence on donor-driven agendas continue to impede evidence-informed decision-making.

Presence of wide variations in the main metrics of economic evaluation studies across and within countries suggests that it may be hard for policymakers to rely on evidence from other settings for decision-making [189]. There is a lack of consensus on which cost effectiveness decision rule or threshold should be applied to determine whether a given intervention is cost effectiveness [190]. This lack of agreement likely explains why studies use different thresholds and reach varying conclusions. Several approaches of determining the thresholds for LMICs, including all African countries have been proposed. For example, the WHO’s CHOICE program [191] popularised the use of thresholds ranging from one to three times GDP per capita while other country specific estimates have been suggested [192,193]. However, most recent estimates that account for resource constraints indicate thresholds below one times GDP per capita, significantly lower than the three times GDP per capita threshold used in many studies. The diversity of decision rules and thresholds further complicates the usability of the economic evidence by policy makers when making resource allocation decisions. It also raises concerns about the affordability of interventions deemed cost-effective under higher thresholds. To enhance the usability of economic evidence in policy formulation and revision, there is a need for an agreed-upon decision rule and cost-effectiveness threshold that reflects local resource constraints. In terms of value for money, vaccines such as rotavirus (Rotarix), malaria (RTS,S), and HPV vaccines generally demonstrated strong economic value across multiple countries.

Finally, the economic evaluation research itself has suffered from important limitations in terms of generating evidence that is timely, relevant, with clear-cut recommendations that meet the needs of policy makers. For example, while economic evaluation metrics such as ICERs convey information about what interventions deliver health benefits at reasonable cost, they do not necessarily address concerns of policymakers who face tight budgetary constraints about whether cost-effective means affordable or even high priority [194]. Even though policy makers could agree on the commendable goals of economic evaluation, many of economic evaluation studies are packaged in highly technical language that is poorly understood by the ultimate consumers of the evidence, thereby lessening their impact on policy [195].

This review has a number of limitations that are important to acknowledge. The first limitation we highlight is that this review included only articles written in English. Secondly, publications not captured in the electronic platforms searched for this study were not included. Just as we excluded unpublished papers. Third, there is limited evidence from the papers whether they were commissioned by national governments in respective countries, or only the funders listed on the papers. These limitations may have limited the scope of the evidence that is available. This being a scoping review, we did not conduct a quality assessment of the included articles so we cannot comment on the quality of the evidence. Our analysis of authorship was limited to the first and last authors of each publication. This approach may have overlooked important collaborations and contributions from middle authors and participating institutions. However, we adopted this strategy because first and last authorship positions are generally regarded as the most influential in health research. In the analysis and comparison of the ICERs as a proportion of the GDP per capita, we did not include the differences in the comparator that may have existed between studies. This presented a generalised picture of the analysis. However, it should be noted that most studies used no vaccine as the comparator. Notwithstanding these limitations, this review provides a starting point for understanding the extent to which economic evaluation studies have been applied to support vaccine policy making in Africa.

## Conclusion

This review identified a sparse but growing literature on economic evidence supply, demand and use in vaccine policymaking in Africa. This review suggests that the challenge facing vaccine economics in Africa is not solely one of evidence generation. Rather, it reflects weaknesses across the entire evidence ecosystem, including the supply of locally relevant economic evidence, the institutional demand for such evidence, and the mechanisms through which evidence is incorporated into policy decisions. Strengthening vaccine policymaking will therefore require simultaneous investments in research capacity, data systems, institutional demand for economic evidence, and formal processes for integrating evidence into decision-making.

## Supporting information

S1 Table Details of the cost effectiveness studies included in the review. (DOCX)

S2 Table Showing the affiliation of the first and last authors and the respective study funders. (DOCX)

S1 Checklist PRISMA Extension for Scoping Reviews Check list as adapted from Tricco et al 2018. (DOCX)
