# Supplementary material for: Supply, demand and use of economic evidence in vaccine policymaking in Africa: A scoping review
Source: PLOS Glob Public Health. 2026 Sep 24;6(9):e0007080. doi: 10.1371/journal.pgph.0007080 (PMC13606984; doi:10.1371/journal.pgph.0007080)
Supplement: Supplementary file 1 [file pgph.0007080.s001.docx]

**S1 Table:** Details of the cost effectiveness studies included in the review

| **Article** | **Intervention (Vaccine/ Vaccination strategy)** | **Comparator** | **Perspective** | **Time horizon** | **Currency year** | **Source of data** | **Model Type used for CEA** | **CE Threshold decision rule** | **CE Threshold used in relation to the decision rule** | **CE Threshold (Amount)** | **ICER for the base case** | **Recommended option** |
| --- | --- | --- | --- | --- | --- | --- | --- | --- | --- | --- | --- | --- |
| Anderson et al 2019^118^ | ETEC and Shigella vaccines | No vaccination | Health system | 10 years | 2016 | Secondary data or literature | cost-effectiveness model | 1-3X GDP per capita | 3x GDP per capita | Various | ETEC: AFRO US$ 2,172 per DALY averted Shigella: AFRO US$ 1,976 per DALY averted | Introducing vaccines only in high burden countries and/regions could reduce cost without substantial reductions in health impact. |
| Anderson et al 2019 | ETEC and Shigella vaccines | Burden without ETEC or Shigella vaccination | Not mentioned | 10 years | 2016 | Secondary data or literature | dynamic models | 0.25 - 4 times GDP | 0.25 to 4X GDP per capita | Not mentioned | All provincial ICERs where above $2000/DALY | considering the subnational variation in ETEC and Shigella burden and access to vaccination when designing vaccination programs may lead to higher impact and improved cost-effectiveness of vaccine deployment programs. |
| Levin et al 2007^65^ | Thermostable versions of four vaccines: (1) measles, (2) yellow fever (YF), (3) bacille Calmette-Gu´ erin (BCG), and (3) diphtheriatetanus–pertussis–hepatitisB(DTP-hepB). | Status quo (existing vaccines for the respective diseases) | Societal | – | 2003 | Secondary data or literature | Decision tree model | 1-3X GDP per capita | 1X GDP per capita | US$960 per DALY averted | Measles: US$36.52 per DALY averted BCG: $812.92 per DALY averted DTP-hep B: $43.63 per DALY averted YF: $2.70 per DALY averted All vaccines (eliminating cold chain costs): US$29.05 per DALY averted | Themosstable vaccines |
| Gargano et al 2017^121^ | Haemophilus influenzae type b-containing and pneumococcal conjugate vaccines | (1) No vaccination, (2–5) one- and two-dose Hibcontaining vaccine or PCV, and (6–7) one-dose or two-doses of combined Hib-containing vaccine | MSF perspective | – | 2013 | Primary data | decision-tree models | 1-3X GDP per capita | 1X GDP per capita | US$1045 per DALY averted | 1 dose Hib: US$ 211 per DALY averted 2 dose Hib: US$ 310 per DALY averted 1 dose PCV: US$ 148 per DALY averted 2 dose PCV: US$ 210 per DALY averted 1 dose each Hib and PCV: US$ 125 per DALY averted 2 doses each Hib and PCV: US$ 2019 per DALY averted | one-dose of either vaccine for children in their first 2 years of life |
| Patterson 2024^115^ | hepatitis A vaccination | No vaccination | Provider | Life time | 2020 | Secondary data or literature | Dynamic Transmission age-structured model | – | – | – | US$ 3276 per DALY averted | implementation of a single dose of the hepatitis Avaccine in South African children < 2 years old between 2023 and 2030 |
| Gosset et al 2021^73^ | HepB-BD + HepB3 | HepB3 | Health system | Lifetime | 2017 | Secondary data or literature l | Markov model | – | 0.5X GDP per capita | US$335.5 per DALY averted | US$18 per DALY averted | implementing HepB-BD in addition to HepB3 in Dafra in the Hauts-Bassins Region of Burkina Faso could be of economic value. |
| Scott et al 2018^82^ | Controlled temperature chain (CTC) strategy for birth dose HBV vaccine delivery | No controlled temperature chain (CTC) HBV vaccine | Health system | Lifetime | 2014 | Secondary data or literature l | Closed cohort model of perinatal HBV transmission and liver disease progression | Not mentioned | Not given | Not given | Sub Saharan Africa: US$1920 per DALYs averted per 1000 births North Africa: US$1500 per DALYs averted per 1000 births | The CTC strategy |
| Griffiths et al 2005^90^ | Hepatitis B vaccine (combination and monovalent vaccine) | No vaccination | Societal | Lifetime | 2001 | Secondary data or literature | – | Comaprison with previous CEA decisions | Comparison of ICER with other CEA results of similar intreventions within similar settings | Not stated | Combined vaccine: US$47 per DALY averted Monovalent vaccine: US$19 per DALY averted | Both options (combined and monovalent vaccines) are cost effective |
| Reardon et al 2019^104^ | –Routine immunization (RI),  –RI plus universal HepB BD,  –RI plus HepB BD only for newborns of hepatitis B surface antigen-positive mothers identified through rapid diagnostic testing (RDT). | Status quo (Routine immunization) | Government | Lifetime | 2015 | Secondary data or literature | Decision tree | 1X GDP per capita | 1X GDP per capita | Djibouti: $3342 per LYS Algeria: $14,613 Per LYS Mauritania: $3835 per LYS | Djibouti: US$0.83 per LYS Algeria: US$1.59 per LYS Mauritania: US$0.18 per LYS | BD plus RI |
| Memirie et al 2020^108^ | Adding a birth dose of HBV vaccine to the current schedule | Status quo (Current practice (pentavalent vaccination (DPT-HiB-HepB) administered at 6, 10 and 14 weeks after birth)) | Provider | 51 years | 2018 | Secondary data or literature | Markov model | 1-3X GDP per capita | 1X GDP per capita | US$2322 per DALY averted | US$110 per DALYs averted | Adding a birth dose of HBV vaccine to the current schedule |
| Kim et al 2007^134^ | HBV vaccine | No vaccination | Societal and payer | Lifetime | 2002 | Secondary data or literature | computer-based model | Societal perspective: compared with past CE decisions Payer perspective: Affordability curves based on past budgets | Societal perspective: 1X per-capita GDP Payer perspective: affordability curve | US$ 300 per DALY averted | Societal perspective: US$ 28 per DALY averted  Payer perspective: US$ 47 per DALY averted | vaccinating infants against HBV |
| Aggarwal et al 2003 | HB vaccination | No vaccination | Societal | 1 year | – | Secondary data or literature | Markov model | – | – | US$440 per QALY gained | US$13.22 per QALY | HB vaccine is eminently suited for inclusionin the national immunization programme of India. |
| Moodley et al 2016^71^ | HIV vaccination | Standard of care | Provider | 70 years | 2012 | Secondary data or literature | Semi-Markov models | – | – | – | US$ 43.07 per QALY | HIV vaccination among adolescents is predicted to result in considerable health benefits and to be cost effective in the South African context. |
| Kiendrebeogo et al 2023 | CERVARIX®,  CECOLIN®,  GARDASIL-4®, and  GARDASIL-9® | No vaccination | Limited societal | Life time | 2022 | Secondary data or literature | UNIVAC decision support model (an Excel based proportionate outcomes static cohort model) | 0.09–0.29 times GDP per capita | 0.09–0.29 times GDP per capita | US$ 266 per DALY averted | CERVARIX®: Dominated CECOLIN®: US$ 242 per DALY averted GARDASIL-4®: Dominated GARDASIL-9®: US$ 988 per DALY averted | CECOLIN® |
| Wondimu et al 2022^26^ | 4vHPV vaccine | 4vHPV vaccine and  No vaccination | Healthcare system | 1 year | 2019 | Secondary data or literature | static Markov cohort model | 1-3X GDP per capita | 3 GDP per capita | US$ 2567 per QALY gained | US$ 454 per QALY gained | 9vHPV vaccine |
| Jit et al 2014^138^ | HPV vaccination | No vaccination | Payer (MoH) | Lifetime | 2011 | Secondary data or literature | Papillomavirus Rapid Interface for Modelling and Economics (PRIME) as a Microsoft Excelbased model that estimates the health and economic eff ect of vaccination of girls against HPV before sexual debut. | 1-3X GDP per capita | 3X GDP per capita | various | various | HPV vaccination |
| Vodicka et al 2022^28^ | Bivalent (Cervarix™) vaccine and  Quadrivalent (Gardasil®) vaccine | No vaccination | Government and societal | Lifetime | 2018 | Secondary data or literature | Proportional outcomes model (UNIVAC, version 1.4) | 4–40% GDP per capita | Atleast 40% of GDP per capita | Atleast 40% of $881 per DALY averted | **Government perspective** Bivalent (Cervarix™) vaccine: $158 per DALY averted Quadrivalent (Gardasil®) vaccine: $494 per DALY averted **Societal perspective** Bivalent (Cervarix™) vaccine: $152 per DALY averted Quadrivalent (Gardasil®) vaccine: $488 per DALY averted | Introducing HPV vaccination |
| Michaeli et al 2022^29^ | Bivalent,  Quadrivalent, and  Nonavalent HPV Vaccination | No vaccination | Public healthcare system | 20 years | 2019 | Secondary data or literature l | Markov model | – | – | – | Bivalent: R−77,115 per QALY gained Quadrivalent: R−72,733 per QALY gained Nonavalent: R−33,908 per QALY gained | nonavalent vaccination is the preferred strategy |
| Mwenda et al 2023^31^ | CECOLIN©,  CERVARIX©,  GARDASIL-4©, and  GARDASIL-9 © | No vaccination and to each other | Government and societal | Lifetime | 2021 | Secondary data or literature | UNIVAC decision-support model, an Excel-based proportionate outcomes static cohort model | – | 0 and 0.5X GDP per capita. | US$1003.5 per DALy averted | **Government perspective** CECOLIN©: $3 per DALY averted CERVARIX©: $42 per DALY averted GARDASIL-4©: $44 per DALY averted GARDASIL-9 ©: $443 per DALY averted **Societal perspective** CECOLIN©: Cost saving CERVARIX©: Cost saving GARDASIL-4©: Cost saving GARDASIL-9 ©: $220 per DALY averted | CECOLIN without cross-protection, or CERVARIX with cross-protection |
| Burger et al 2018^44^ | One-dose HPV vaccination | Two-dose HPV vaccination and No vaccination | Not mentioned (Likely societal perspective) | Life time | 2011 | Secondary data or literature | Three-tiered hybrid modeling approach: 1) dynamic agent-based model of HPV transmission (‘‘Harvard-HPV”), 2) static individual-based model of cervical carcinogenesis (‘‘Harvard-CC”) and 3) companion population-based model (‘‘Harvard-Scale Up”). | 1-3X GDP per capita | 1X GDP per capita | US$1603 per DALY averted | Scenario A (1 dose vs no vaccine): Cost saving Scenario B (1 dose vs 2 doses): I$ 111 per DALY averted | Routine one-dose HPV vaccination is better in comparison to no vaccination Two dose HPV vaccination is better when compared to 1 dose HPV vaccination |
| Li et al 2015^53^ | AS04-adjuvanted HPV-16/18 vaccine in combination with current cytological screening | Cervical cancer screening alone | Public payer | Lifetime | 2013 | Secondary data or literature | Markov | 1-3X GDP per capita | 3X GDP per capita | ZAR 200,292 per QALY gained | ZAR 81 978 per QALY gained | AS04-adjuvanted HPV-16/18 vaccine in combination with current cytological screening |
| Campos et al 2012^60^ | (1) HPV 16/18 vaccination of pre-adolescent girls;  (2) screening of adult women over age 30; and  (3) pre-adolescent vaccination followed by screening at older ages. | Screening only | Not mentioned | Life time | 2005 | Secondary data or literature | – | 1-3X GDP per capita | 3 GDP per capita | Kenya (I$4410); Mozambique (I$2373); Tanzania (I$3501); Uganda (I$3231) per YLS | Kenya: I$ 2090 per LYS Mozambique:I$ 1260 per LYS Tanzania: I$ 740 per LYS Uganda: I$ 1000 per LYS | Combination of HPV vaccination and Screening |
| Drolet et al 2021^17^ | 27 HPV vaccination strategies varying the age of routine vaccination (9 years or 14 years), number of age cohorts vaccinated, the population targeted (girls only or girls and boys), and the interval between doses | No vaccination | Payer | 100 years | 2017 | Secondary data or literature | Mathematical dynamic model | – | Both 1X GDP per capita and 0·5X GDP per capita | Uganda: I$1864 and I$932; and Nigeria: I$5861 and I$2930 per DALYs averted | Uganda I$ 89 per DALYs averted Nigeria I$ 550 per DALYs averted | Routine vaccination of girls aged 9 years, with additional vaccination of girls aged 9–14 years in the first year of the programme, |
| Ekwunife & Lhachimi 2017^88^ | Current scenario of opportunistic screening (CS) National screening using VIA (NS) National HPV vaccination in addition to opportunistic screening (CS + NV) National screening and national HPV vaccination (NS+NV) | No vaccination | Provider | Lifetime | 2015 | Secondary data or literature | Markov model | 1-3X GDP per capita | 3 GDP per capita | US$ 9610 per DALYs averted | US$ 7930 DALY averted | National HPV vaccination in addition to opportunistic screening (CS + NV) |
| Sinanovic et al 2009^97^ | HPV vaccination of 12-year-old girls followed by screening for cervical cancer | Status quo (Current strategy within the National Cervical Cancer Screening Programme for diagnosis of cervical disease) | Societal | Lifetime | 2007 | Secondary data or literature | Static Markov state transition model | 1-3X GDP per capita | 3 GDP per capita | US$ 17172 per QALY gained | $1460 per QALY gained | HPV vaccination with screening |
| Tracy et al 2014^98^ | HPV vaccination | No vaccination | Not mentioned | 40 years | 2011 | Secondary data or literature | Compartmental mathematical models [compartmental (susceptible-infected-recovered [SIR]) model] | 1-3X GDP per capita | 3X GDP per capita | I$ 3600 per LYS | I$ 1178 per life year saved | HPV vaccination with screening |
| Goldie et al 2008^103^ | HPV 16,18 vaccination | No vaccination | Societal | 10 years | 2005 | Secondary data or literature | The companion model (micro-simulation model) based on the structure of a decsion tree | 1-3X GDP per capita | 3X GDP per capita | Numerous | Numerous | HPV vaccination |
| Kiatpongsan & Kim 2014^128^ | 9-valent vaccine | Current (2-or 4-valent) vaccines in Kenya and Uganda | Societal | – | 2005 | Secondary data or literature | natural history disease simulation model of HPV and cervical cancer | 1-3X GDP per capita | Both 1x per-capita GDP and 3x per-capita GDP | Kenya: I$1470 and Uganda I$1077 | Not mentioned | 9-valent vaccine remains cost-effective compared to vaccination |
| Guimarães et al 2023^129^ | CECOLIN,  CERVARIX, and  GARDASIL-4 | No vaccination | Government | 10 years | 2021 | Secondary data or literature | UNIVACdecision-support model (version 1.54) | 1X GDP per capita | 1X GDP per capita | 35% of US$ 500 per DALY averted | **With Gavi Funding** Cecolin: $2.5 per DALY averted GARDASIL-4: $2.8 per DALY averted CERVARIX: $3.5 per DALY averted  **Without Gavi Funding** Cecolin: $27 per DALY averted GARDASIL-4: $43 per DALY averted CERVARIX: $52 per DALY averted | HPV vaccination is a cost-effective intervention in Mozambique. |
| Schmit et al 2024^32^ | R21/Matrix-M | A range of malaria transmission settings | Government | 15 years | 2023 | Secondary data or literature | semi-mechanistic model | – | – | – | $19–68 per DALY averted | introducing R21/Matrix-M into routine childhood immunisation in malaria-endemic areas of sub-Saharan Africa |
| Bell et al 2020^49^ | Malaria vaccine (RTS,S/AS01) and bed nets | No vaccination and no bed nets | Not mentioned | 3 years | Not mentioned | Primary data | – | Not mentioned | Not mentioned | – | Lilongwe Urban: US$38·91 cases averted Lilongwe Rural: US$16·43 cases averted | Malaria Vaccine |
| Galactionova et al 2017^61^ | RTS,S/AS01 vaccine given in four doses | No vaccination | Societal | 10 years | 2013 | Secondary data or literature | A micro-simulation model of malaria epidemiology and control | 1-3X GDP per capita | 1X GDP per capita | US$ 842 per DALY averted | US$188 per DALYs averted | RTS,S/AS01 vaccine given in four doses |
| Seo et al 2014^66^ | RTS,S vaccines- malaria vaccine | Delivering LLINs to children | Societal | 60 years | 2012 | Secondary data or literature | Markov model | 1-3X GDP per capita | 1X GDP per capita | US$ 1095 per DALY averted | Societal perspective: US$ 91.18 per DALY averted Provider perspective: US$ 145.03 per DALY averted | Malaria Vaccine |
| Penny et al 2016^19^ | RTS,S/AS01 malaria vaccine | No vaccination | Provider | 15 years | 2013 | Secondary data or literature | 4 models | – | – | – | Three dose schedule: $2 per dose (ICER: $35 per DALYs averted ) $5 per dose (ICER: $80 ) $10 per dose (ICER: $147 ) Four dose schedule: $2 per dose (ICER: $38 per DALYs averted ) $5 per dose (ICER: $87 ) $10 per dose (ICER: $154 ) | Not mentioned in the paper |
| Topazian et al 2023^20^ | RTS,S malaria vaccine | Long-lasting insecticide-treated nets (LLINs) Indoor residual spraying (IRS) Seasonal malaria chemoprevention (SMC) | Provider | 15 years | 2015 | Secondary data or literature | four mathematical models | Not mentioned | Not mentioned | Not mentioned | Not mentioned | Long-lasting insecticide-treated nets (LLINs) |
| Sauboin et al 2019^110^ | –Child vaccination (doses at ages 6, 7.5, 9, and 27 months) with RTS,S Malaria Vaccine –Infant vaccination (doses at ages 6, 10, and 14 weeks and 21 months) with RTS,S Malaria Vaccine | No vaccination | Health system | 15 years | 2015 | Secondary data or literature | Static Markov cohort model | 1-3X GDP per capita | 1X GDP per capita | Various | Child vaccinated: US$ 200 per DALY averted  Infant vaccinated: US$ 225 per DALY averted | Child immunisation |
| Ndeketa 2020^114^ | RTS,S vaccine | No vaccination | Provider, household and societal | 15 years | Not mentioned | Secondary data or literature | Markov static cohort model | 1-3X GDP per capita | 1X GDP per capita | US$ 398.6 per DALY averted | **Healthcare system perspective**  at US$ 1 per dose : ICER $40 per DALY averted at US$ 5 per dose : ICER $115 per DALY averted at US$ 10 per dose : ICER $209 per DALY averted **Societal perspective**  at US$ 1 per dose : ICER $34 per DALY averted at US$ 5 per dose : ICER $109 per DALY averted at US$ 10 per dose : ICER $202 per DALY averted | Introduction of the RTS,S/AS01 vaccine would be a highly cost-effective malaria intervention in Malawi. |
| Byberg et al 2017^50^ | "MV for all policy", Measles Vaccination of children at health facility regardless of the number and age of children eligible for measles vaccination available. | Status quo (a local policy where MV vials are only opened if six or more children 9–11 months of age are present at the same time (‘restrictive MV policy’)) | Societal | 36 months | Not mentioned | Primary data | – | Not mentioned | Not mentioned | Not mentioned | US$ 5.61 per discounted LYG | MV for all policy |
| Lee et al 2019^56^ | 2-dose measles immunization outreach strategy for geographically hard-to-reach target populations | No outreach | Not mentioned | 5 years | 2018 | Secondary data or literature | susceptible-exposed-infectious-recovered (SEIR) compartment model | 1-3X GDP per capita | 1X GDP per capita | US$ 1865 per DALY averted | various | 2-dose measles immunization outreach strategy for geographically hard-to-reach target populations |
| Wedlock et al 2019^76^ | Measles-rubella (MR) vaccine | Baseline (no threshold, 10-dose MCVs) | Health systems | 1 year | 2018 | Secondary data or literature | HERMES-generated supply chain model | 1-3X GDP per capita | 1X GDP per capita | Benin: US$829.8  Niger: US$378.1 Mozambique: Us$415.7 per DALY averted | Benin: scenario 6: US$ 471.10 per DALY averted Niger: scenario 9: US$ 36.62 per DALY averted Mozambique: scenario 9: US$ 16.05 per DALY averted | Benin: scenario 6, Using 5-dose vials of MCV and opening them when 60% of children to utilise the doses are available  Niger: scenario 9, Using mixed dose vials of MCV and opening them when 60% of children to utilise the doses are available Mozambique: scenario 9,Using mixed dose vials of MCV and opening them when 60% of children to utilise the doses are available |
| Doshi et al 2017^79^ | Strategy 2: One dose of measles vaccine plus national SIAs up to the age of five years.  Strategy 3: Two doses of measles vaccine delivered through routine immunization services at 9 months and 18 months of age. | Receiving one MCV during routine immunization (One dose of measles vaccine delivered through the routine immunization services at 9 months of age at the most recent reported coverage rate.) | Provider | 15 years | 2015 | Secondary data or literature | an age structured SIR (Susceptible-Infectious-Recovered) model | – | – | – | Strategy 3: $ -1144 per death averted Strategy 2: $-1275 per death averted | Strategy 3: Two doses of measles vaccine delivered through routine immunization services at 9 months and 18 months of age |
| Kaucley & Levy 2015^107^ | Combination of Routine Immunization program (RI) and Supplementary Immunization Activities (SIAs) for delivery of the measles vaccination | No vaccination | Provider | 1 year | 2011 | Primary data | – | Comaprison with previous CEA decisions | Comparison with other vaccines that have been recommended (measles vaccine) | – | US$3 per DALY averted | Combination of routine and supplementary measles vaccination strategies |
| Doshi et al 2015^123^ | Measles Containing Vaccine (MCV) | One dose of MCV through routine immunization services | Provider | 15 years | – | Secondary data or literature | age-structured SIR (Susceptible-Infectious-Recovered) model | – | – | – | Strategy 1 (one dose at 9 to11 month sold): Ref Strategy 2 (MCV1 through RI services, MCV2 through SIA): US$ 1275 per death averted Strategy 3 (two doses): US$ 1144 per death averted | Incorporate the second dose of MCV intothe routine immunization program |
| Bishai et al 2011^124^ | Supplemental Immunization Activities (SIAs) | No SIAs scenario | Societal | 40 years | 2010 | Secondary data or literature | dynamic stochastic model | – | – | – | US$1.50 per DALY averted | SIAs are a more cost-effective approach to produce health than are other interventions currently supported. |
| Zimmermann et al 2019^133^ | measles containing vaccine (MCV) | Northern regions | Vaccination program and health system | 10 years | 2018 | Secondary data or literature | generic branch of EMOD, stochastic agent-based model | – | – | – | – | we found that the current strategy of nationwide SIAs every 2 years in Nigeria is sub-optimal. Increasing SIA frequency in Northern regions (where RI coverage is low and birth rates are high) and decreasing frequency in the South (where RI coverage is higher and birth rates are lower) could significantly reduce measles incidence, and in some cases reduce the total vaccination cost |
| Arifin et al 2019^45^ | Four vaccination strategies that differ by the types of vaccines used (MenAfriVac™, PMP, and PMC) | The base strategy is Routine (EPI) MenAfriVac™ at 9 months and reactive (during outbreak) PMP vaccine for 1–29 years | Payer | 28 years | 2016 | Secondary data or literature | Agent-based model (ABM) of meningococcal transmission | 1-3X GDP per capita | 3X GDP per capita | US$ 3231 per DALY averted | Base prime strategy: US$ 662 per DALY averted Prevention 1: US$ 2,437 per DALY averted Prevention 2: US$ 2900 per DALY averted | Base prime strategy that is routine (EPI) PMC vaccine at 9 month and reactive PMC vaccine for 1-29 years |
| Kuznik et al 2017^94^ | Quadrivalent conjugate (serogroup ACYW conjugate) vaccination against Neisseria meningitidis | Monovalent serogroup A vaccination | Health system | 40 years | 2014 | Secondary data or literature | Markov model | 1-3X GDP per capita | 1X GDP per capita | various | Range from US$105 DALY averted in Ghana to $252 DALY averted in Chad | expanding routine coverage from the monovalent serogroup A vaccine to broader coverage with a quadrivalent ACWY vaccine would provide good value per dollar spent even at incidence rates that fall well below the WHO epidemic threshold |
| Pugh et al 2019^119^ | Pneumococcal Conjugate Vaccine (10- or 13-Valent) | No vaccination | Payer | 1 year | 2017 | Secondary data or literature | A decision-analytic model | 1-3X GDP per capita | 1X GDP per capita | Not mentioned | Algeria: US$ 475 per QALY gained Tunisia: US$ 1239 per QALY gained | both PCV10 and PCV13 are cost effective strategies in both Algeria and Tunisia. |
| Touray et al 2011^142^ | Pneumococcal vaccination | No vaccination | Societal | 5 years | 2000 | Secondary data or literature | Decision tree | 1-3X GDP per capita | 1X GDP per capita | Various | I$100 per DALY averted | Pneumococcal vaccination |
| Sibak et al 2015^22^ | 13-valent pneumococcal conjugate vaccine (PCV13), using a three-dose schedule without booster | No vaccination | Government | 5 years | 2013 | Secondary data or literature | TRIVAC model (version 2.0) | 1-3X GDP per capita | 3X GDP per capita |  | US$ 3916 per DALY averted | the 13-valent pneumococcal conjugate vaccine (PCV13) |
| Sevilla et al 2022^27^ | PCV13 and  PCV10 NVIs | PCV13,  PCV10 NVIs and  No vaccination | health payer and societal perspectives | 100 years | 2016 | Secondary data or literature | NA | 1-3X GDP per capita | 1 GDP per capita | US$ 3,479 per QALY gained | PCV10: $1,984.41 per QALY gained PCV13: $925.60 per QALY gainedr PCV13 relative to PCV10: $174 QALY gained | PCV13 |
| Pecenka et al 2021^38^ | PCV vaccination | No vaccination | Government and societal | 5 years | 2017 | Secondary data or literature | The UNIVAC model framework (V.1.3.7) | – | 15% of per capita GNI | 15% of US$750 per DALY averted | Government perspective: US$91 per DALY averted Societal perspective: US$56 per DALY averted | PCV may be a better buy than indicated by previous economic studies undertaken in The Gambia and other low- income countries |
| Kebede et al 2019^43^ | Universal vaccination program | No universal vaccination program | Societal |  | 2013 | Primaty data | quasi-experimental evaluation | 1-3X GDP per capita | 1X GDP per capita | US$ 505 per DALY averted | US$ 281.4 per DALY averted | 10- valent pneumococcal vaccination |
| Kim et al 2010^51^ | PCV7,  PCV9,  PCV10, and  PCV13 | No vaccination | Societal | 5 years | 2005 | Secondary data or literature | Static, aggregate-level, state-transition model | 1-3X GDP per capita | 3X GDP per capita | US$ 1080 per DALY averted | US$ 670 per DALY averted | PCV 9 |
| Ojal et al 2019^63^ | Pneumococcal conjugate vaccine continuation beyond 2022 when Kenya was expected to graduate from Gavi funding | Stopping vaccination in year 2022 | Societal | 15 years | 2016 | Secondary data or literature | Dynamic pneumococcal trans mission model in combination with a costing model | 1-3X GDP per capita | 1X GDP per capita | US$ 1455 per DALY averted | US$153 per DALY averted US$952 per case averted US$6856 per death averted | Continuation of PCV |
| Ibrahim et al 2024^69^ | PCV13 vaccination programme beyond the GAVI transition (2026 to 2031) and  PCV13 vaccination programme in 2012 to 2025 | No vaccination | Government | 5 years | 2022 | Secondary data or literature | UNIVAC(version 1.4) model | 1-3X GDP per capita | 1X GDP per capita | USD 2445.3 per DALY averted | US$ 510 DALYs averted | PCV13 vaccination programme post Gavi |
| Ayieko et al 2013^80^ | 10-valent vaccine (PCV10) 13-valent vaccine (PCV13) | No vaccination | Societal | 5 years | 2010 | Secondary data or literature | A static decision analytic model | 1-3X GDP per capita | 1X GDP per capita | US$ 775 per DALY averted | PCV10: US$59 per DALY averted PCV13: US$47 per DALY averted | PCV10 |
| Sinha et al 2007^135^ | pneumococcal vaccination | No vaccination | Societal | – | 2000 | Secondary data or literature | decision analysis model | 1-3X GDP per capita | Various | Various | US$101 per DALY averted | at a price of $5 per dose, pneumococcal vaccine would be a highly cost-eff ective purchase in 68 of the 72 GAVI-eligible countries, on the basis of WHO standards for assessment of the economic value of health interventions. |
| Griffiths et al 2006^85^ | Nine polio vaccination alternatives of inactivated poliovirus vaccine (IPV) instead of oral poliovirus vaccine (OPV) | 6 doses of oral poliovirus vaccine (OPV) | Societal | Lifetime | 2005 | Secondary data or literature | – |  | Not mentioned | Not mentioned | 2 doses IPV in 10 dose vial: $61,355 per DALY averted 3 doses IPV in single dose vial: $158,884 per DALY averted 3 doses IPV in 10 dose vial: $117,822 per DALY averted All remaining ICERs were above $150,000 per DALY averted | 6 doses of OPV |
| Laufer et al 2023^113^ | RSV LRTI prevention strategies | No RSV LRTI prevention | Government and donor payer | 3 years | 2019 | Secondary data or literature | – | – | – | – | US$ 891 per DALY averted | RSV LRTI prevention |
| Guimarães et al 2022 | ROTARIX,  ROTAVAC ROTASIIL | No vaccination | Government | 15 years | 2018 | Secondary data or literature | Universal vaccine costeffectiveness and impact modelling framework (UNIVAC) proportionate outcomes decision-support model | 0.5X GDP per capita | 0.5X GDP per capita | US$ 224 per DALY averted | **With Gavi support** Rotarix 2016-2020: US$ 70 per DALY averted Rotarix 2021-2030: US$ 102 per DALY averted Rotavac: US$ 122 per DALY averted Rotasill: US$ 121 per DALY averted **Without Gavi support** Rotarix 2016-2020: – Rotarix 2021-2030: US$ 330 per DALY averted Rotavac: US$ 295 per DALY averted Rotasill: US$ 259 per DALY averted | ROTARIX |
| Debellut et al 2022^34^ | ROTARIX, ROTAVAC, and  ROTASIIL | No vaccination and to each other | Government and societal | 10 years | 2021 | Primary data | Excel proportionate outcomes model, UNIVAC (V.1.4.30) | – | 0.5X GDP per capita | US$ 277 per DALY averted | **Government Perspective** ROTAVAC: US$76 per DALY averted ROTASIIL: US$104 per DALY averted ROTARIX: US$146 per DALY averted **Societal perspective** ROTAVAC: US$72 per DALY averted ROTASIIL: US$99 per DALY averted  ROTARIX: US$141 per DALY averted | ROTAVAC is likely the most cost- effective option. |
| Nonvignon et al 2018^36^ | Rotavirus vaccination | No vaccination | Healthcare system and societal | 20 years | 2015 | Secondary data or literature | TRIVAC model | 1-3X GDP per capita | 1X GDP per capita | US$1328 per DALY averted | **Scenario 1** Government perspective: US$249 per DALY averted Societal perspective: US$238 per DALY averted **Scenario 2** Government perspective: US$344 per DALY averted Societal perspective: US$332 per DALY averted | Rotavirus vaccination is highly cost-effective |
| Sigei et al 2015^37^ | RV1 introduction | No vaccination | Government and societal | 20 years | 2016 | Secondary data or literature | TRIVAC cost-effectiveness model (version 2.0) | 1-3X GDP per capita | 3x GDP per capita | – | Kenya: $38 per DALY averted  Uganda: $34 and US$ 29 per DALY averted | rotavirus vaccine introduction is cost-effective |
| Janko et al 2022^41^ | monovalent,  pentavalent, or  both | No vaccination | Payer and health sector | Lifetime | 2017 | Secondary data or literature | Bayesian mixed-effects meta-regression framework | 1-3X GDP per capita | Various | Various | various | Introducing rotavirus vaccination worldwide |
| Abbott et al 2012^52^ | Rotavirus vaccine (RotaTeq) | No vaccination | Health provider | 5 years | 2009 | Primary data | – | 1-3X GDP per capita | 1X GDP per capita | US$ 695 per DALY averted | US$ 62.26 per DALY averted | RotaTeq vaccine |
| Niyibitegeka et al 2021^57^ | Rotarix®,  RotaTeq®,  Rotavac® and  Rotasiil® | No vaccination | Societal | 5 years | 2019 | Primary data | Markov model | 1-3X GDP per capita | 1X GDP per capita | US$ 378 per DALY averted | Rotarix: $93 per DALYs averted RotaTeq: $263 per DALYs averted Rotavac: $69 per DALYs averted Rotasiil: $124 per DALYs averted | All vaccines |
| Diop et al 2015^58^ | Rota virus vaccine | No vaccination | Societal | 20 years | 2013 | Secondary data or literature | The TRIVAC model, developed by researchers from the London School of Hygiene and Tropical Medicine (LSHTM) | 1-3X GDP per capita | 3 GDP per capita | US$ 1040 per DALY averted | Provider perspective: $92 per DALY averted Societal perspective:$73 per DALY averted | Rotavirus vaccine |
| Ortega et al 2009^64^ | Rotarix (GlaxoSmithKline) vaccine | No vaccination | Government | 5 years | 2005 | Secondary data or literature | Decision tree model | – | – | – | US$ 363 per DALYs averted | Rotarix vaccine |
| Bar-Zeev et al 2016^67^ | Monovalent rotavirus vaccination | No vaccination | Societal | 20 years | 2014 | Primary data |  | 1-3X GDP per capita | 1X GDP per capita | US$ 759 per DALY averted | Societal perspective: US$ 10 per DALY averted Provider perspective: US$ 19 per DALY averted | Monovalent rotavirus vaccination |
| Mohy et al 2024^68^ | HBRV (RotaTeq, Merck, USA) and BRV-PV 1dose vial (Rotasiil 1-dose vial, Serum Institute of India, India) | HRV (Rotarix, GSK, Belgium) | Societal | 1 year | 2022 | Secondary data or literature | A model was developed in Microsoft Excel | 1-3X GDP per capita | 1X GDP per capita | US$ 3500 per DALY averted | HBRV: US$ -591,274.9 per QALY gained BRV-PV_1d: US$ -26,674.6 per QALY gained | HRV (Rotarix, GSK, Belgium) |
| Alkoshi et al 2014^70^ | Rotavirus vaccination | No vaccination and vaccinated situations | Healthcare providers and societal | 5 years | 2012 | Primary data | Disease-specific decision tree model | 1-3X GDP per capita | 1X GDP per capita | US$10,132 per QALY gained | US$8,972 per QALY gained both pespectives | Introduction of rotavirus vaccination is expected to alleviate a significant burden of the disease and is highly cost-effective in Libya. |
| Verguet et al 2013^72^ | rotavirus vaccination | No vaccination | Household | 20 years | – | Secondary data or literature | ECEA approach | – | – | – | Not mentioned | importance of vaccine pricing, specifically GAVI co-financing. |
| Berry et al 2010^75^ | Rotarix vaccine | No rotavirus vaccination | Not mentioned | 2 years | 2008 | Secondary data or literature | – | 1-3X GDP per capita | 1X GDP per capita | US$312 per DALY averted | US$74.73 per DALY averted | adding Rotarix vaccination to the existing vaccine infrastructure in Malawi would likely be a highly cost-effective intervention |
| Anderson et al 2020^78^ | General rotavirus vaccine | No vaccination | Government | 5 years | 2019 | Secondary data or literature | Deterministic model developed in Microsoft Excel | – | – | – | US$ 47 per DALY averted | general rotavirus vaccine |
| Pecenka et al 2018^84^ | Rotarix ,  ROTAVAC , and  ROTASIIL | No vaccination | Payer | 5 years | Not mentioned | Secondary data or literature | TRIVAC model, a static cohort model developed at the LondonSchoolof Hygieneand Tropical Medicine with support from the Pan American Health Organization’s ProVac Initiative. | GNI per capita | 0.3 times GNI per capita | Ghana: US$1380 per DALY averted Malawi:US $320 per DALY averted | **Cost/DALY averted with/without Gavi subsidy** Ghana: Rotarix: US$230/$312, ROTAVAC: $283/$360, ROTASIIL: $358/$479 Malawi: Rotarix: US$7/$241, ROTAVAC: $38/$250, ROTASIIL: $32/$392 | Rotarix |
| Van Hoek et al 2012^95^ | Rotarix vaccine and  RotaTeq vaccine | No vaccination | Societal | 5 years | 2011 | Secondary data or literature | – | 1-3X GDP per capita | 1X GDP per capita | US$ 1600 per DALY averted | Rotarix: US$ 142 per DALY averted RotaTeq: US$ 288 per DALY averted | Rotarix vaccine |
| Gargano et al 2015^109^ | 1- Adding rotavirus vaccine to the routine immunization program (RI)  2- Adding rotavirus vaccine to the national supplemental vaccination campaign (SIA) | No vaccination | Provider | 1 year | 2011 | Secondary data or literature | Decision tree models | 1-3X GDP per capita | 1X GDP per capita | Us$336 per DALY averted | RI: US$5.30 per DALY averted SIA: US$37.62 per DALY averted | Both interventions RI and SIA for rotavirus vaccination are cost effective |
| Ruhago et al 2015^112^ | –Management of diarrhoea –The current treatment of diarrhoea (using oral rehydration salt (ORS) and intravenous (IV) fluid), with the addition of rotavirus vaccination to the current diarrhoea treatment  –Provision of rotavirus vaccine (RV1) alone | No vaccination | Provider | 5 years | 2012 | Primary data | Markov model | 1-3X GDP per capita | 1X GDP per capita | US $609 per DALY averted | US$ 112 per DALY averted | RV1 vaccine added to current diarrhoea treatment |
| Tate et al 2009^127^ | rotavirus immunization program | No vaccination | Societal | 2 years | 2007 | Secondary data or literature | Microsoft Excel–based model | 1-3X GDP per capita | 1X GDP per capita | US$1740 per DALY averted | $166 per DALY | introduction of a program would still be considered to be a highly cost-effective intervention at higher prices of $7.35, $9.26, and $20.31 per course |
| Wenge 2024^136^ | 1) – Two doses of Rotarix administered at 6 and 10 weeks (current strategy) 2) –Three doses of Rotarix administered at 6, 10, and 14 weeks  3) –Three doses of Rotarix administered at 6, 10, and 40 weeks  4) –Three doses of the neonatal RV3-BB vaccine administered at 1, 6, and 10 weeks | No vaccination | Government and societal | 10 years | 2025 | Secondary data or literature | Decision tree | 1-3X GDP per capita | 0.5X GDP per capita | US$ 335 per DALY averted | US$45.89 per DALY averted | Neonatal RV3-BB vaccine |
| Kim et al 2011^93^ | -Rotavirus vaccine -HPV vaccine | No vaccination | Societal | Rotavirus: 5 years HPV: Lifetime | 2005 | Secondary data or literature | Static cohort model (implicitly based on a decision tree) | 1-3X GDP per capita | 1X GDP per capita | various | various | Rotavirus vaccine and HPV |
| Tate et al 2011^48^ | pneumococcal and  rotavirus vaccination programs | No vaccination | Public healthcare system | – | 2008 | Secondary data or literature | Decision tree | 1-3X GDP per capita | 1X GDP per capita |  | Rota virus: US$3.96 per DALY averted Pneumococcal pneumonia: US$-37 per DALY averted | Both vaccines are recommended |
| Anderson et al 2023^137^ | Shigella vaccination | No vaccination | Health system | 20 years | 2019 | Secondary data or literature | Simulation model | Comaprison with previous CEA decisions | Comparison with other vaccines that have been recommended | – | Not mentioned | potential Shigella vaccine could have a considerable public health benefit |
| Channing and Sinanovic 2014^106^ | New strategy in which infants receive BCG and a booster vaccine, MVA85A, at 4 months of age. | Status quo (existing strategy of BCG vaccination at birth) | Government | 10 years | 2012 | Secondary data or literature | Markov model | Not mentioned | Not stated | Not stated | US$1,105 per TB case averted | Existing strategy of BCG vaccination at birth |
| Phillips et al 2023^30^ | Typhoid conjugate vaccines (TCVs) | No vaccination | Healthcare system perspective | 10 years | 2020 | Secondary data or literature | age-specific stochastic model | – | – | – | $300 per DALY averted | TCVs with a catch-up campaign |
| Antillón et al 2017^23^ | Five TCV delivery strategies: (I) routine vaccination at 9months of age; and routine vaccination at 9months plus a one-time catch-up campaign among individuals (II) 9 months to 5 years old, (III) 9 months to 15 years old, (IV) 9 months to 25 years old, (V) all ages 9months. | No vaccination | Payer | 10 years | 2015 | Secondary data or literature | Age-stratified compartmental model of typhoid transmission (‘‘transmission model”) and added a probability model describing treatment outcomes (‘‘treatment model”) | 1-3X GDP per capita | Both 1X GDP per capita and 3X GDP per capita | I$ 3082.50 per DALY averted | Nairobi: I$ 2390 per DALY averted  Lwak: I$ 6931 per DALY averted | Routine infant vaccination alone (intervention I) |
| Carias et al 2015^77^ | ViCPS vaccine | No vaccination | Public healthcare | 3 years | 2014 | Secondary data or literature | static model | 1-3X GDP per capita | 1X GDP per capita | US$ 598 per DALY averted | US$ 484 per DALY averted | a one-time fixed-post typhoid vaccination campaign in Kasese District, Uganda, was estimated to be a highly cost-effective intervention |
