# Supplementary material for: Supply, demand and use of economic evidence in vaccine policymaking in Africa: A scoping review
Source: PLOS Glob Public Health. 2026 Sep 24;6(9):e0007080. doi: 10.1371/journal.pgph.0007080 (PMC13606984; doi:10.1371/journal.pgph.0007080)
Supplement: Supplementary file 2 [file pgph.0007080.s002.docx]

**S2 Table:** Showing the affiliation of the first and last authors and the respective study funders

| **Article** | **Country(ies) of study** | **Country of affiliation of 1st author** | **Country of affiliation of last author** | **Funder** |
| --- | --- | --- | --- | --- |
| Guimarães et al 2022 | Mozambique | Mozambique and Portugal | Switzerland | Bill and Melinda Gates Foundation |
| Kiendrebeogo et al 2023 | Burkina Faso | Burkina Faso and Germany | United Kingdom | PATH |
| Wondimu et al 2022^26^ | Ethiopia | Netherlands and Ethiopia | Netherlands | Netherlands Organization for International Cooperation in Higher Education |
| Sevilla et al 2022 | Egypt | United States | United States | Pfizer Inc |
| Vodicka et al 2022 | Ghana | United States | United States | PATH, Gavi, the Vaccine Alliance, or the Bill & Melinda Gates Foundation |
| Michaeli et al 2022 | South Africa | Germany and United kingdom | Germany | Projekt DEAL |
| Phillips et al 2023 | Malawi | United States | United States | Bill and Melinda Gates Foundation |
| Mwenda et al 2023 | Kenya | Kenya | United Kingdom | German Federal Ministry of Education and Research |
| Schmit et al 2024 | Burkina Faso | United Kingdom | United Kingdom | The Wellcome Trust, the Bill & Melinda Gates Foundation, the UK Medical Research Council, the European and Developing Countries Clinical Trials Partnership 2 and 3, the NIHR Oxford Biomedical Research Centre, and the Serum Institute of India, Open Philanthropy |
| Mohy et al 2023 | South Africa | Belgium | Argentina | GlaxoSmithKline Biologicals SA |
| Debellut et al 2022 | Niger | Switzerland | France and United States | Bill & Melinda Gates Foundation |
| Okafor & Ekwunife 2021 | Central African Republic, Chad, Comoros, Equatorial Guinea, Gabon, Guinea, Somalia, and South Sudan | Australia | Nigeria | Copenhagen Consensus Center USA, and the Bill & Melinda Gates Foundation |
| Nonvignon et al 2018 | Ghana | Ghana | Ghana | Bill & Melinda Gates Foundation |
| Sigei et al 2015 | Kenya and Uganda | Kenya | United Kingdom | – |
| Pecenka et al 2021 | Gambia | United States | Gambia and United Kingdom | Gavi, The Vaccine Alliance; Bill & Melinda Gates Foundation; MRC (UK); Pfizer |
| Hidle et al 2018 | Zimbabwe | United States | Zimbabwe | Gavi, The Vaccine Alliance and the United States Centers for Disease Control and Prevention |
| Hsiao et al 2023 | Tanzania | Germany | Germany | UK Department of Health and Social Care (DHSC), the Foreign, Commonwealth & Development Office (FCDO), the Global Challenges Research Fund (GCRF), the Medical Research Council (MRC), Wellcome, and the Bill and Melinda Gates Foundation |
| Janko et al 2022 | 195 countries | United States | United States | Bill and Melinda Gates Foundation |
| Soi et al 2019 | Mozambique | United States | United States | Gavi, the Vaccine Alliance Brocher Foundation |
| Kebede et al 2019 | Ethiopia | Sweden | Sweden | Swedish Research Council |
| Burger et al 2018 | Uganda | United States and Norway | United States | Bill & Melinda Gates Foundation |
| Arifin et al 2019 | Niger | United States | United States | PATH German Research Foundation (DFG) |
| Simuyemba et al 2024 | Zambia | Zambia | Zambia | U.S. National Institutes of Health |
| Hidle et al 2022 | Zimbabwe | United States | United States | Gavi, the Vaccine Alliance |
| Tate et al 2011 | Uganda | United States | Ethiopia | – |
| Bell et al 2020 | Malawi | United States | United States | – |
| Byberg et al 2017 | Guinea-Bissau | Guinea-Bissau and Denmark | United Kingdom | Danish Council for Independent Research Danish National Research Foundation European Union |
| Antillón et al 2017 | Kenya | United States | United States | Bill and Melinda Gates Foundation and Research Foundation – Flanders (FWO) |
| Kim et al 2010 | Gambia | United States | United States | Bill and Melinda Gates Foundation |
| Abbott et al 2012 | Ghana | United States | United States | – |
| Li et al 2015 | South Africa | Belgium | Belgium | GlaxoSmithKline Biologicals S.A. |
| Debellut et al 2022 | Malawi | Switzerland | United States | Bill & Melinda Gates Foundation |
| Mak et al 2024 | Zambia | United States | United States | Bill and Melinda Gates Foundation |
| Lee et al 2019 | Kenya | united States | United states | Centers for Disease Control and Prevention (CDC) the International Society for Infectious Diseases (ISID) and Pfizer |
| Niyibitegeka et al 2021 | Burundi | Thailand | Thailand | Thailand International Cooperation Agency (TICA) |
| Diop et al 2015 | Senegal | Senegal | Senegal | Bill and Melinda Gates Foundation |
| Byberg et al 2017 | Guinea-Bissau | Guinea-Bissau and Denmark | United Kingdom | Danish Council for Independent Research, DANIDA, European Union |
| Campos et al 2012 | Kenya, Mozambique, Tanzania, Uganda, and Zimbabwe | United States | United States | Bill & Melinda Gates Foundation |
| Drolet et al 2021 | Uganda, and Nigeria | Canada | Canada and United Kingdom | Bill & Melinda Gates Foundation |
| Tseng et al 2011 | Zambia | Canada | Canada | Canadian Institutes of Health Research |
| Galactionova et al 2017 | 43 African countries | Switzerland | Switzerland | PATH Malaria Vaccine Initiative (MVI) and the Bill and Melinda Gates Foundation (BMGF) |
| Ngabo et al 2015 | Rwanda | Rwanda and Belgium | Switzerland | – |
| Ojal et al 2019 | Kenya | Kenya and United kingdom | United Kingdom | Wellcome Trust and the Royal Society |
| Ortega et al 2009 | Egypt | United States | Egypt | None |
| Levin et al 2007 | Ghana | United States | United States | Bill & Melinda Gates Foundation |
| Seo et al 2014 | Malawi | United Kingdom | United Kingdom | None |
| Bar-Zeev et al 2016 | Malawi | Malawi and United Kingdom | United Kingdom | Wellcome Trust |
| Mohy et al 2024 | Morocco | Belgium | Morocco | GlaxoSmithKline Biologicals SA |
| Ibrahim et al 2024 | Ghana | Ghana | Ghana | None |
| Alkoshi et al 2014 | Libya |  | Malaysia | Ministry of Higher Education, Malaysia |
| Moodley et al 2016 | South Africa | South Africa and Switzerland | South Africa and Switzerland | National Institute of Allergy and Infectious Diseases (NIAID) U.S. Public Health Service Grants |
| Verguet et al 2013 | Ethiopia | United States | United States | Bill & Melinda Gates Foundation |
| Gosset et al 2021 | Burkina Faso | France | France | Total Foundation |
| Baral et al 2021 | Kenya and Malawi | United States | United States | Bill & Melinda Gates Foundation |
| Berry et al 2010 | Malawi | United States | United States | National Center for Research Resources |
| Wedlock et al 2019 | Benin, Mozambique and Niger | United States | United States | Bill and Melinda Gates Foundation National Institute of General Medical Sciences Modeling Infectious Disease Agent Study (MIDAS) |
| Carias et al 2015 | Uganda | United States | United States | CDC Global Disease Detection Operations Center Outbreak Response Contingency Fund |
| Anderson et al 2020 | Nigeria | United States | United States | Bill and Melinda Gates Foundation |
| Doshi et al 2017 | DRC | United States | United States | None |
| Ayieko et al 2013 | Kenya | Kenya | United Kingdom | GAVI Alliance and Wellcome Trust |
| Levin et al 2013 | Uganda | United States | United States | Bill & Melinda Gates Foundation |
| Scott et al 2018 | Global | Australia | Australia | Burnet Institute. |
| Usuf et al 2014 | Gambia | Gambia and United Kingdom | United Kingdom | Global Alliance for Vaccines and Immunisation (GAVI) and PATH |
| Pecenka et al 2018 | Ghana and Malawi | United States | United kingdom | Bill & Melinda Gates Foundation |
| Griffiths et al 2006 | South Africa | United Kingdom | South Africa | WHO, Geneva, Switzerland |
| Dayan et al 2004 | Zambia | United States | United States | – |
| Griffiths et al 2016 | Zambia | United Kingdom | South Africa | Bill & Melinda Gates Foundation |
| Ekwunife & Lhachimi 2017 | Nigeria | Germany and Nigeria | Germany | Alexander von Humboldt Foundation (AVH) |
| Madsen et al 2014 | Malawi | Denmark | Denmark | – |
| Griffiths et al 2005 | Mozambique | Switzerland | Mozambique | – |
| Slavkovsky et al 2024 | Ethiopia, Rwanda, and Uganda | United States | Uganda | Bill and Melinda Gates Foundation |
| Galactionovan et al 2015 | Burkina Faso, Ghana, Kenya, Senegal, Tanzania, and Uganda | Switzerland | Switzerland | Bill and Melinda Gates Foundation and PATH |
| Kim et al 2011 | 72 GAVI-eligiblecountries | United States | United States | Bill and Melinda Gates Foundation and Harvard Global Health Institute |
| Kuznik et al 2017 | 26 countries of the African meningitis belt. | United States, Nigeria and Uganda | Nigeria | Bayero University, Kano, Nigeria |
| Van Hoek et al 2012 | Kenya | United Kingdom | Kenya and United Kingdom | None |
| Quentin et al 2012 | Tanzania | Germany | Tanzania and United Kingdom | World Health Organization, the Wellcome Trust and a Cervical Cancer Initiative grant from the Union of International Cancer Control (UICC). |
| Sinanovic et al 2009 | South Africa | South Africa | South Africa | Bill & Melinda Gates Foundation. |
| Hutubessy et al 2012 | Tanzania | Switzerland | Switzerland | GAVI Alliance and the Bill and Melinda Gates Foundation |
| Tracy et al 2014 | Mali | United States | United States | – |
| Vaughan et al 2020 | Tanzania | United States | United States | Bill & Melinda Gates Foundation, Seattle, WA |
| Griffiths et al 2009 | Ethiopia | United Kingdom | Ethiopia | – |
| Alonso et al 2019 | Mozambique | United Kingdom | Mozambique | NA/GAVI Alliance |
| Penny et al 2016 | Ethiopia | Switzerland | United kingdom | PATH Malaria Vaccine Initiative; Bill & Melinda Gates Foundation; Global Good Fund; Medical Research Council; UK Department for International Development; GAVI, the Vaccine Alliance; WHO. |
| Topazian et al 2023 | Sub Saharan Africa | United Kingdom | United Kingdom | Global Fund |
| Haidari et al 2017 | Mozambique | United States | United states | International Society for Infectious Diseases (ISID) and Pfizer |
| Baral et al 2023 | Ghana, Kenya, and Malawi | United States | United states | World Health Organization |
| Goldie et al 2008 | 72 GAVI-eligible countries | United States | United states | Bill and Melinda Gates Foundation |
| Reardon et al 2019 | African region | United States | United states | Duke Emergency Medicine Global Health Grant |
| Sicuri et al 2019 | Burkina Faso, Ghana, Kenya, Mozambique, and Tanzania | Spain | Belgium | GlaxoSmithKline Biologicals S.A. |
| Channing and Sinanovic 2014 | South Africa | South Africa | South Africa | Oxford-Emergent Tuberculosis Consortium (OETC) |
| Kaucley & Levy 2015 | Benin | Benin | France | Priority Solidarity Fund of French Ministry of Foreign Affairs through the Agence de Médecine Préventive (AMP) |
| Memirie et al 2020 | Ethiopia | Ethiopia and Norway | United States | Bill & Melinda Gates Foundation |
| Gargano et al 2015 | Somalia | United States | United States | – |
| Sauboin et al 2019 | 41 SSA countries | Belgium | Belgium | GlaxoSmithKline Biologicals S.A. |
| Kananura et al 2024 | Uganda | Kenya and Uganda | United States | Bill and Melinda Gates Foundation |
| Ruhago et al 2015 | Tanzania | Norway | Norway | Norwegian State Education Loan Fund (Statens Lånekasse) and the University of Bergen, Norway, |
| Laufer et al 2023 | Mali | United States | United States | Bill & Melinda Gates Foundation |
| Ndeketa 2020 | Malawi | Malawi | United Kingdom | Wellcome Trust and Bill and Melinda Gates Foundation |
| Patterson 2024 | South Africa | South Africa | South Africa and United Kingdom | Department of Science and Innova tion/National Research Foundation |
| Mvundura 2024 | Ethiopia | United States | United States | Bill and Melinda Gates Foundation to PATH |
| Ndikumukiza 2021 | Rwanda | United States | Saudi Arabia | None |
| Anderson et al 2019 | 79 low- and lower middle-income countries | United States | United States | PATH from the Bill & Melinda Gates Foundation |
| Pugh et al 2019 | Tunisia and Algeria | United States | United States | Pfizer |
| Colombini et al 2015 | Burkina Faso | Switzerland | France | Bill & Melinda Gates Foundation |
| Gargano et al 2017 | South Sudan | United States | United States | – |
| Anderson 2019 | Democratic Republic of Congo (DRC), Kenya, Zambia, and Zimbabwe | United States | United States | PATH from the Bill & Melinda Gates Foundation |
| Doshi et al 2015 | Democratic Republic of Congo | United States | United States | None |
| Bishai et al 2011 | Uganda | United States | Switzerland | World Health Organization and the Bill and Melinda Gates Foundation. |
| Jeuland et al 2009 | Mozambique | United States | United States | Bill & Melinda Gates Foundation |
| Diawara et al 2023 | Mali and Burkina Faso | Mali | United States | Bill and Melinda Gates Foundation |
| Tate et al 2009 | Kenya | United States | United States | – |
| Kiatpongsan & Kim 2014 | Kenya and Uganda | Thailand | United States | Bill and Melinda Gates Foundation |
| Sibak et al 2015 | Egypt | Egypt | France | Bill and Melinda Gates Foundation and The Agence de Médecine Préventive (AMP) |
| Guimarães et al 2023 | Mozambique | Mozambique and Portugal | United Kingdom | Bill and Melinda Gates Foundation and PATH |
| Levin et al 2011 | Bangladesh, Brazil, Colombia, Ethiopia, and Uganda | United States | Switzerland | World Health Organization |
| Ilboudo et al 2022 | Benin | Burkina Faso | Burkina Faso | Pfizer Innovative Health Global Vaccines |
| Brennan et al 2022 | Senegal | United States | United States | Gavi, the Vaccine Alliance |
| Zimmermann et al 2019 | Nigeria | United States | United States | Bill and Melinda Gates Foundation |
| Kim et al 2007 | Gambia | United States | United States | Harvard Graduate Society Fellowship |
| Sinha et al 2007 | 72 countries | United States | United States | Gavi, the Vaccine Alliance |
| Wenge 2024 | Malawi | United States | United States | US National Institutes of Health/National Institute of Allergy and Infectious Diseases, the Wellcome Trust , Bill and Melinda Gates Foundation, and US Centres for Disease Control and Prevention, and the UK National Institute for Health and Care Research (NIHR) |
| Anderson et al 2023 | 102 low-income and middle-income countries | United States | Belgium | Bill & Melinda Gates Foundation and Wellcome Trust. |
| Jit et al 2014 | 179 countries | United Kingdom | Switzerland | WHO |
| Levin et al 2014 | India, Peru, Uganda, Vietnam, and Tanzania | United States | Switzerland | World Health Organization |
| Debellut et al 2024 | Malawi | Switzerland | United States | Bill & Melinda Gates Foundation |
| Mvundura et al 2023 | Ethiopia, Guyana, Rwanda, Senegal, Sri Lanka, and Uganda | United States | United States | Bill & Melinda Gates Foundation |
| Touray et al 2011 | Gambia | United Kingdom | Switzerland | None |
