# Supplementary material for: Supply, demand and use of economic evidence in vaccine policymaking in Africa: A scoping review
Source: PLOS Glob Public Health. 2026 Sep 24;6(9):e0007080. doi: 10.1371/journal.pgph.0007080 (PMC13606984; doi:10.1371/journal.pgph.0007080)
Supplement: Supplementary file 4 [file pgph.0007080.s005.docx]

**Point-by-point reviewers’ comments and authors’ answers**

Title of the manuscript: “Supply, demand and use of economic evidence in vaccine policymaking in Africa:

A scoping review”

| **#** | **Editorial and reviewers’ comments** | **Authors’ response** | **Line & page No.** |
| --- | --- | --- | --- |
| **#** | **Editorial comments** | **Authors’ response** |  |
| **1** | **Journal requirements:**  Please amend your detailed Financial Disclosure statement. This is published with the article. It must therefore be completed in full sentences and contain the exact wording you wish to be published.  i. Please clarify all sources of financial support for your study. List the grants, grant numbers, and organizations that funded your study, including funding received from your institution. Please note that suppliers of material support, including research materials, should be recognized in the Acknowledgements section rather than in the Financial Disclosure.  ii. State the initials, alongside each funding source, of each author to receive each grant. For example: "This work was supported by the National Institutes of Health (####### to AM; ###### to CJ) and the National Science Foundation (###### to AM)."  iii. State what role the funders took in the study. If the funders had no role in your study, please state: “The funders had no role in study design, data collection and analysis, decision to publish, or preparation of the manuscript.”  iv. If any authors received a salary from any of your funders, please state which authors and which funders. | We thank the reviewer for this important comment. We have modified the text as follows:  “This study is part of the Enhancing the use of Economic Evidence in Vaccine Policy Making in Africa project that was supported by the Wellcome Trust, grant number WCT-EVP01. The funder had no role in the study design, data collection and analysis, decision to publish, or preparation of the manuscript.” | Page 31, Lines 904-908 |
| **2** | We note that your Data Availability Statement is currently as follows: All data used in the analysis have been provided as part of the submitted article.    Please confirm at this time whether or not your submission contains all raw data required to replicate the results of your study. Authors must share the “minimal data set” for their submission. PLOS defines the minimal data set to consist of the data required to replicate all study findings reported in the article, as well as related metadata and methods (<https://journals.plos.org/plosone/s/data-availability#loc-minimal-data-set-definition>).    For example, authors should submit the following data:    - The values behind the means, standard deviations and other measures reported;  - The values used to build graphs;  - The points extracted from images for analysis.    Authors do not need to submit their entire data set if only a portion of the data was used in the reported study.    If your submission does not contain these data, please either upload them as Supporting Information files or deposit them to a stable, public repository and provide us with the relevant URLs, DOIs, or accession numbers. For a list of recommended repositories, please see <https://journals.plos.org/plosone/s/recommended-repositories>.    If there are ethical or legal restrictions on sharing a de-identified data set, please explain them in detail (e.g., data contain potentially sensitive information, data are owned by a third-party organization, etc.) and who has imposed them (e.g., an ethics committee). Please also provide contact information for a data access committee, ethics committee, or other institutional body to which data requests may be sent. If data are owned by a third party, please indicate how others may request data access. | Thank you for the comment. Yes, we confirm that all data needed to replicate the study findings have been shared. | NA |
| **3** | Some material included in your submission may be copyrighted. According to PLOS’s copyright policy, authors who use figures or other material (e.g., graphics, clipart, maps) from another author or copyright holder must demonstrate or obtain permission to publish this material under the Creative Commons Attribution 4.0 International (CC BY 4.0) License used by PLOS journals. Please closely review the details of PLOS’s copyright requirements here: PLOS Licenses and Copyright. If you need to request permissions from a copyright holder, you may use PLOS's Copyright Content Permission form.  Please respond directly to this email or email the journal office and provide any known details concerning your material's license terms and permissions required for reuse, even if you have not yet obtained copyright permissions or are unsure of your material's copyright compatibility.  Potential Copyright Issues:  a. Figure 2: please (a) provide a direct link to the base layer of the map (i.e., the country or region border shape) and ensure this is also included in the figure legend; and (b) provide a link to the terms of use / license information for the base layer image or shapefile. We cannot publish proprietary or copyrighted maps (e.g. Google Maps, Mapquest) and the terms of use for your map base layer must be compatible with our CC-BY 4.0 license.  Note: if you created the map in a software program like R or ArcGIS, please locate and indicate the source of the basemap shapefile onto which data has been plotted.  If your map was obtained from a copyrighted source please amend the figure so that the base map used is from an openly available source. Alternatively, please provide explicit written permission from the copyright holder granting you the right to publish the material under our CC-BY 4.0 license.  Please note that the following CC BY licenses are compatible with PLOS license: CC BY 4.0, CC BY 2.0 and CC BY 3.0, meanwhile such licenses as CC BY-ND 3.0 and others are not compatible due to additional restrictions.  If you are unsure whether you can use a map or not, please do reach out and we will be able to help you. The following websites are good examples of where you can source open access or public domain maps:  * U.S. Geological Survey (USGS) - All maps are in the public domain. ([http://www.usgs.gov](http://www.usgs.gov/))  * PlaniGlobe - All maps are published under a Creative Commons license so please cite “PlaniGlobe, [http://www.planiglobe.com](http://www.planiglobe.com/), CC BY 2.0” in the image credit after the caption. (<http://www.planiglobe.com/?lang=enl>)  * Natural Earth - All maps are public domain. (<http://www.naturalearthdata.com/about/terms-of-use/>) | The Figure was developed with Microsoft Excel.  We have now included the following text in the figure notes.  “This map was created with Microsoft Excel.” | Page 15, Lines XX |
| **#** | **Reviewer 1 comments** | **Authors’ response** |  |
| **4** | **Introduction**  The introduction is not very aligned with the focus of the review and should be rewritten. It is not clear there is a direct linkage between disease burden and generation or use of evidence for decision making. If there is, the authors have not made it clear. | Once again, we are grateful for this comment. We have edited the introduction section to align more with the focus of the review. In particular, we have highlighted the importance of disease burden influencing priority setting or vaccine policy-making. Some of the text we have included are as below.  “Currently, little is known about how national immunization programmes prioritize new vaccine introductions and vaccine delivery strategies. A scoping review by Guillaume et al found that much of the evidence used in priority-setting regarding vaccines is dominated by burden of disease metrics such as the number of illnesses, deaths or disability-adjusted life-years averted. Other considerations in the common criteria included vaccine price and financing.^12^. However, immunisation policy makers and planners in LMICs generally lack information on the costs and cost-effectiveness analysis of immunisation interventions^19^.” | Page 4, Lines 194-198 |
| **5** | The authors have not adequately captured other vaccine-related scoping reviews that exist. There are some that are included in their selected articles but these should be mentioned in the introduction and it should be clear on how this review builds off the prior review and what the limitations of the vaccine costing or CEA reviews done to date are. | Thanks for the comment. We now captured two scoping reviews that we found relevant and explained the available literature enabling us to identify the available gap. We have modified the text as follows;  “Currently, little is known about how national immunization programmes prioritize new vaccine introductions and vaccine delivery strategies. A scoping review by Guillaume et al found that policymakers in LMICs focus so much on the disease targeted by the vaccine in the priority setting of new vaccines. Some of the common criteria considered included disease burden, cost of disease while the economic criteria used included vaccine price, affordability and financing^11^. However, immunisation policy makers and planners in LMICs generally lack information on the costs and effectiveness of immunisation interventions^19^. Despite the general understanding that the health impact of most childhood interventions is strong, the cost-effectiveness of various immunization interventions in various contexts remains unclear^19,20^. A review by Anosike et al reported the cost effectiveness and budget impact of vaccines targeting any of the three diseases that is malaria, measles and meningitis in Africa found that there was limited evidence on cost effectiveness of the vaccines in Africa^21^. However, the Anosike et al study was limited to only three diseases, did not address the challenges of economic evidence generation and use in policy making in Africa. Addressing these knowledge gaps is increasingly important as African countries assume greater responsibility for financing immunization programmes, strengthen HTA and evidence-informed decision-making processes, and seek to maximise health gains from limited public resources.” | Pages 4&5, Lines 194-228 |
| **6** | The introduction has some statements that seem subjective. For example, the statement on page 3 that reads “Currently, little is known about how immunization programs prioritize new vaccine introductions and vaccine delivery strategies.” seems subjective. We know prioritization is done at the global level through WHO and Gavi and some countries also have NITAGs to inform the introduction decision. Please provide validation for your statement here. | We agree with the reviewer on this point. To acknowledge this point, we have included details on institutions that have stated or been reported to conduct some priority setting, while highlighting the importance of national programmes in using local evidence to support their decisions. We have included the following text.  “The growing importance of economic evidence has coincided with efforts to strengthen institutional mechanisms for evidence-informed vaccine decision-making. At the global level, the World Health Organization supports evidence-based vaccine recommendations through the Strategic Advisory Group of Experts on Immunization (SAGE), while Gavi has promoted the use of evidence in decisions regarding vaccine introduction and programme financing and strengthening countries’ prioritisation of vaccines appropriate to their context as one of its strategic objectives^5^. At the national level, some countries have established National Immunization Technical Advisory Groups (NITAGs) to provide independent recommendations based on epidemiological, economic, programmatic, and contextual evidence^16,17^. In parallel, an increasing number of African countries are institutionalising Health Technology Assessment (HTA) as a mechanism to improve transparency, efficiency, and accountability in health sector priority-setting and resource allocation^18^. Together, these developments have created growing demand for high-quality, locally relevant economic evidence to support vaccine policy decisions.”  We have slightly modified the statement “Currently, little is known about how immunization programmes prioritize new vaccine introductions and vaccine delivery strategies.” Because based on the available literature, we are convinced that it is a statement of fact especially at national level. We have specified the above statement to focus on national level as below.  “Currently, little is known about how national immunization programmes prioritize new vaccine introductions and vaccine delivery strategies.” | Page 4, Lines 167-179  Page 4, Lines 194-195 |
| **7** | **Methods:**  On page 4, what do the authors mean when they said they included studies of all study designs? Later in the PRISMA flow chart, there is mention that 7 studies were excluded because of wrong study design. This seems contradictory. Please explain what studies designs were in and out of scope. | Thank you for the comment. We excluded studies of systematic or scoping review design and editorials. We have made this clear in the text below.  “We included studies of all study designs except editorials, and reviews (systematic and scoping reviews).” | Page 5, Lines 257-258 |
| **8** | The paragraph on page 6 that starts with “ we searched PubMed…” duplicates what was stated on page 4 last paragraph. | Thank you for the observation. We have deleted the repetition. | Page 7 |
| **9** | The selection of sources of evidence needs to be more specific. Can you provide initials of the authors who were involved in the steps included such as assessing abstracts, full text and also resolving conflicts of classification.  In the section on Data Items, please separate data items related to costing from those related to CEAs as these are different. | Thank you for this comment. We have added the initials of the independent researchers that did the screening and full text reading as below.  “The uploaded articles were assessed for similarity and duplicates removed. Screening of titles and abstracts of uploaded articles was done by two independent researchers, EM and JK. This was followed by full text review to assess the articles for inclusion into the study based on the inclusion and exclusion criteria. In the event of any conflicts on whether an article fulfils the inclusion criteria, a third researcher (FM) made the final decision.”  “Some of the data items that were captured from all included economic evaluation studies are author, year of publication, aim of the study, vaccine under consideration, study design, country where the study was conducted, study population, sample size, type of study (e.g. cost effectiveness analysis, cost utility, costing), model used, comparator, currency and year, costing perspective, administration strategy, costs, other key findings, funding source, conflict of interest. Data items extracted from economic evaluations other than costing studies include outcome measures, time horizon, cost effectiveness threshold used, ICER, cost effectiveness decision. For studies that focused on the use of economic evidence in decision making, we extracted data on author, year of publication, aim of the study, study design, country where the study was conducted, existing structures utilising economic evidence for decision making, challenges and enablers of economic evidence use for decision making among others.” | Page 7, Lines 307-311  Page 7&8, Lines 316-339 |
| **10** | The authors mention that they asses “…existing structures utilizing economic evidence for decision making, challenges and enablers of economic evidence use for decision making…”. How was this done? This is one specific objective of this manuscript, but it is not clear how this information was assessed as the manuscripts were being reviewed. | Thanks for the comment. We have provided additional details as below. “Existing structures using economic evidence were identified as such if a study explicitly reports it for example stating whether NITAG or an HTA agency used economic evidence.” | Page 7&8, Lines 326-339 |
| **11** | **Findings**  Can the authors provide more information on why articles were excluded from the reviews. This is especially true for the 4037 studies screened, why were 3821 excluded. Similarity, of those selected for retrieval, why were 7 not retrieved? | Thank you for the comment. Articles were removed following the title and abstract screening because they were deemed not relevant based on the inclusion and exclusion criteria. It is standard practice that a reason is not provided for each excluded article at that stage. However, reasons are provided for articles that progress to full text reading and excluded at that stage.  The 7 articles were not retrieved because their full texts couldn’t be found. These may have been studies presented at conferences and without full text versions published. | Page 09 |
| **12** | As mentioned before, the methods say all study designs were included but the flow chart shows 7 were rejected for wrong study design. Please clarify. | Yes, and thank you for pointing this out. We have addressed this comment above. | NA |
| **13** | Page 9 mentions study participants, should this be target population? | Thank you for the observation. Yes, it should be study population. We have replaced it in text as follows:  “Regarding study populations, the most commonly reported study population were children under five years (n=38 studies; 29.5%) followed by newborns (n=8 studies; 6.2%), girls aged nine years (n=7 studies; 5.4%), and infants (n=6 studies; 4.7%).” | Page 10, Lines 451-454 |
| **14** | May you also add information on whether the studies had local collaborators and if any of the authors were government staff. | Thank you for the suggestion. For this study, we focused on the first and last authors. We are afraid that the suggestion goes beyond the scope of this study. It is a good suggestion for us to consider in future studies. | NA |
| **15** | For table 3, I would suggest the authors separate costing studies from CEAs as the data extraction information is different. It would remove the blanks that are in the table. Also, there is no clear order for Table 3 as it is neither alphabetical (by author, country) and not y year of publication. Please revise the table for better ordering. In Table 3, why do you include the column on CEA threshold decision used and threshold applied as these are very similar. | Thank you for the comment. We have spilt table 3 into three separate tables to cater for the different types of methods used.  We have also arranged the contents of the table based on the alphabetical order of the authors.  We have included columns for the decision rule used and the CE threshold applied. | Pages 12-17  Tables 3-5 |
| **16** | The table seems to include reviews as well. Some information seems incorrect. For example, there is an article attributed to Slavkovsky et al. 2024 and mentions the country focus as Ethiopia, Rwanda and Uganda. But that article was a systematic review. Can you also identify the other review included in your extraction and whether the actual source articles reviewed are included. | Slavkovsky et al. 2024 study is a modelling study of five countries of which 3 are African countries. The authors use available literature to populate the models. It is not a systematic review otherwise; it would have been excluded. | Pages 16 &17  Table 4 |
| **17** | The map on page 15 is not very informative. It could be improved by stating within each country, how many studies were done. | We are grateful for this suggestion. We have added the number of studies within the map. | Page 19,  Figure 2 |
| **18** | I am surprised to see NGOs such as PATH mentioned as funders as these do not fund studies. | Thank you for the observation. We extracted information as reported by the authors. PATH was mentioned by several studies as a funder. | Page 22, Figure 5 |
| **19** | Table 4 is hard to follow and understand.  You provide a synthesis of the CEAs but there is no similar synthesis for costing studies. What is the rationale for this? | Thank you for the comment. We agree that based on the title of table 6 (updated), it was unclear. We have made the title clearer as below.  “Table 6: The proportion of studies categorized by the ICER of vaccine interventions as a percentage of the GDP per capita in each cost effectiveness perspective”  We did not do a similar synthesis for the costing studies because we focused on ICERs of which costing studies do not have ICERs. | Page 24 |
| **20** | How did the authors determine whether the studies informed policy?  There is mention on page 22 of qualitative studies. Where any insights provided from the quantitative studies on the use of evidence for decision making? What criteria or information were you looking for in the CEAs or costing studies that gave insights into whether the evidence generated was used for decision making? | We thank the reviewer for this comment. We determined that evidence was used if the study explicitly mentioned it. This is why it is based on the qualitative studies. For CEA, the institution commissioning the study and stating the reason why could indicate chances of use of the evidence for example if it is Ministry of Health that has commissioned the study then, it may be likely that such results are used. However, due to the speculative nature of such an approach, re resorted to reporting what was explicitly stated by the authors. | Page 27 |
| **21** | Did the authors do a critical appraisal of the quality of the evidence? | No, we did not conduct a critical appraisal of quality because this is a scoping review. It is not mandatory nor common for scoping reviews to undertake critical appraisal of the quality of evidence. However, we restricted our scoping review to peer-reviewed Journal articles. | NA |
| **22** | Also was there a review of how the studies adhered to costing or CEA reporting standards such as CHEERs? | We did not review studies in relation to the CHEERS reporting guidelines because it was beyond the scope of this study. | NA |
| **23** | **Discussion:**  Statements such as “It is hard for policy makers to rely on evidence from other settings for decision making...” seem subjective. This review does not seem to have done a critical appraisal of the existing evidence and so some of these statements made in the discussion are hard to justify, | Thank you for the comment. We have rephased the statement as below to clearly show that it is an explanation and implication of our findings backed up by other literature.  “Presence of wide variations in the main metrics of economic evaluation studies across and within countries suggests that it may be hard for policymakers to rely on evidence from other settings for decision-making^166.”^ | Page 30, Lines 813-815 |
| **24** | There are several other subjective statements in the discussion section. An example is on page 23 which reads “Economic evidence has not been formally adopted as a requirement for vaccine policy in Africa”. This seems subjective as there are some countries that are requiring evidence as part of UHC. Also, some NITAGs do use this evidence for decision making. | We agree with the reviewer that this point needs to be nuanced. We agree that economic evidence is being used for some vaccine related decision making as we have acknowledged in both the introduction and discussion. However, based on the volume of published literature that we have documented, it can be argued that officially, it is not mandatory to use economic evidence although it is encouraged. We have updated the text as follows:  “Although economic evaluation is encouraged to be used for vaccine decision making^5,6,17,174^, it has not been formally adopted as a requirement for vaccine policy in Africa^16^.” | Page 29, Lines 781-783 |
| **25** | There is also an allusion that “…provided countries with free vaccines…”. There are no free vaccines as countries always have copayments and with Gavi 6.0 the payments are now even more. | Thank you for the comment. We agree and have modified the statement to the following.  “About 48% of vaccine budgets in Africa is funded by donors, notably Gavi, UNICEF, WHO, Gates Foundation, and others, who have provided countries with vaccines, technical support, vaccine introduction grants, etc^175^.” | Page 29, Lines 784-788 |
| **26** | Also, Gavi through the VIS process had used evidence from global CEAs for decision making. | We have acknowledged Gavi in the introduction and discussion section. For example, we have included the following text in the discussion section.   “Recently, Gavi and WHO have encouraged countries to deliberately integrate economic evidence into vaccine policy through the establishment of NITAGs at country level and Gavi has itself used economic evidence in its vaccine investment strategy (VIS) process^174^.” | Page 29, Lines 790-796 |
| **27** | There were also several initiatives such as PROVAC to inform vaccine introduction decisions that should be acknowledged. That PROVAC work lead further development of models such as UNIVAC to enable CEAs. All these efforts have not been acknowledged by the authors and yet it is relevant context. | We have modified the text as follows, “African governments should promote the utilization of already established tools that can speed up generation of economic evidence at a less cost for example the UNIVAC and COSTVAC tools used for estimation of cost effectiveness and vaccine cost respectively that have been developed by the Pan American Health^180^. | Page 28, Lines 701-709 |
| **28** | There is also lack of mention of the lack of country specific CEA thresholds and the lack of cross vaccine CEAs as most CEAs compare vaccine to no vaccine. | Thank you for comment. We discussed the thresholds considerably. In addition, we have added information on the no vaccine comparison as below. “The scarcity of context-specific data likely constrained the evaluation of multiple policy alternatives, leading most studies to assess vaccine interventions against a no-intervention comparator.” | Page 29, Lines 767-771 |
| **#** | **Reviewer 2 comments** | **Authors’ response** |  |
| **29** | Strengthen the overall framing. At the moment, the paper feels like a compilation of studies rather than a clear argument. You present the evidence well, but it’s not organised around a strong central lens. It would help to structure the analysis more clearly, for example by thinking in terms of the supply of economic evidence, the demand for it, and how systems actually use or fail to use it. That would give the paper more direction. | We thank you for the insightful suggestion. We agree that the manuscript was initially structured primarily as a descriptive synthesis of studies. To strengthen the analytical framing, we reorganized the discussion sections around a "supply-demand-use" framing of economic evidence. We now discuss the findings in terms of (i) the supply of economic evidence, including its quantity, geographical distribution, authorship, funding sources, (ii) the demand for economic evidence, reflected by policy requirements, and decision-making structures; and (iii) the use of economic evidence in vaccine policy processes, including enabling factors and barriers to evidence uptake. We hope this provides a clearer narrative linking evidence generation, demand, and utilization in vaccine policymaking across Africa. | Pages 28-30 |
| **30** | The section on use of evidence needs more depth. This is probably the most important part of the paper, but it is currently quite thin. You note that economic evidence is used in a limited way, mostly for costing and planning. But the paper doesn’t really unpack how decisions are made in practice, or the role that different actors, especially donors versus governments, play in shaping those decisions. This section needs to go further and give a clearer picture of what is actually happening on the ground. | Thank you for the comment. We agree that understanding how economic evidence is used in practice is an important area of inquiry. However, the available literature identified through this review provides only limited empirical evidence on actual vaccine decision-making processes. Of the eleven studies included in the "use of economic evidence" component of the review, the majority explored stakeholder perceptions and reported barriers and facilitators to evidence use, while only a small number described actual decision-making processes. We have therefore been cautious not to draw conclusions beyond what is supported by the available evidence. | NA |
| **31** | Clarify the link to policy-making. There is a bit of a mismatch between the title and the content. The title suggests a focus on policy-making, but most of the analysis is about the availability of studies. It would help to be more explicit about the difference between evidence existing, evidence being used, and evidence actually influencing decisions. If the conclusion is that influence is limited, that should be stated clearly and framed as a key finding. | We have modified the title to make it more aligned with the content of the paper. The title has been updated to: “Supply, demand and use of economic evidence in vaccine policymaking in Africa:  A scoping review” | Page 1, Lines 1-2 |
| **32** | Move beyond description and add interpretation. The results are detailed, but they often stop at reporting patterns without explaining them. For example, HPV and rotavirus dominate the literature, but the paper doesn’t explore why. Similarly, the concentration of studies in a few countries is noted but not really interpreted. Throughout the results section, you can strengthen the paper by briefly explaining what these patterns likely mean. | Thank you for raising this point. We have included explanations of patterns for example we have included the following:  “The concentration of studies on HPV and rotavirus vaccines likely reflects a combination of disease burden, donor priorities, and policy interest. Both vaccines have received substantial support from Gavi and other global partners over the past two decades and have been the subject of extensive vaccine introduction and sustainability discussions^5^. Consequently, countries and development partners have had strong incentives to generate evidence on their affordability, implementation costs, and value for money.” | Pages 28, Lines 708-713 |
| **33** | Strengthen the discussion on cost-effectiveness thresholds. You correctly highlight the widespread use of the 1–3 times GDP per capita threshold, but the discussion doesn’t really engage with the ongoing debate around this. It would help to briefly touch on issues like affordability versus cost-effectiveness, and the shift towards opportunity cost-based thresholds. This is an important part of the economic evaluation discussion and would strengthen the paper. | We have built on the discussion for example,  “However, most recent estimates that account for resource constraints indicate thresholds below one times GDP per capita, significantly lower than the three times GDP per capita threshold used in many studies. The diversity of decision rules and thresholds further complicates the usability of the economic evidence by policy makers when making resource allocation decisions. It also raises concerns about the affordability of interventions deemed cost-effective under higher thresholds. To enhance the usability of economic evidence in policy formulation and revision, there is a need for an agreed-upon decision rule and cost-effectiveness threshold that reflects local resource constraints.” | Page 30, Lines 820-825 |
| **34** | Make the policy implications more concrete. The discussion identifies important barriers such as limited funding, weak data systems, and lack of institutional structures. However, the recommendations remain quite general.  It would improve the paper to be more specific about what should change, for example in terms of institutionalising HTA, strengthening data systems, or aligning donor processes with national systems. | We have addressed this by providing more specific recommendations for example in the texts below.  “African governments should promote the utilization of already established tools that can speed up generation of economic evidence at a less cost for example the UNIVAC and COSTVAC tools used for estimation of cost effectiveness and vaccine cost respectively that have been developed by the Pan American Health^180^.”  “There is a growing need to institutionalise HTA as part of routine policy and priority-setting processes. Strengthening HTA systems could enhance the generation, appraisal, and use of economic evidence, reduce reliance on ad hoc decision-making, and support transparent and sustainable vaccine investment decisions in resource-constrained settings.” | Pages 28, Lines 701-707  Pages 30, Lines 799-803 |
| **35** | Simplify and better synthesise the ICER findings. The ICER results are detailed but not easy to interpret at a glance. The key messages are there, but they are buried in the tables. In the text, it would help to clearly state the main takeaways, such as which vaccines consistently show strong value for money, and how results change when Gavi support is included. | Thank you for the comment. We have highlighted this as below. “Overall, vaccines consistently exhibit a good value for money. The value for money increases to the countries when Gavi support is considered.” | Page 23, Lines 626-628 |
| **36** | Minor Comments: There are a few small issues to clean up. For example, “Meta-Analyse” should be corrected to “Meta-Analysis.” Some sentences are quite long and could be tightened for clarity. The rationale for excluding COVID-19 vaccines could also be stated more clearly. The large tables are useful but might be better placed in the supplementary materials, with only key summaries in the main text. It would also help to keep terminology consistent throughout the paper. | Thank you for the comment. We have reviewed the paper and made the suggested changes. For example,  “We included studies that report on cost and economic evaluations of any vaccine administered to children and youth except COVID vaccines. The vaccines targeting COVID 19 were excluded because they are currently not meant for routine administration and are majorly targeting adults. In addition, we included studies report on the use of economic evidence in decision making in any country in Africa”  We have broken the big tables into smaller tables as suggested by reviewer 1. We hope that is okay. | Pages 5-30 |
