# Supplementary material for: Supply, demand and use of economic evidence in vaccine policymaking in Africa: A scoping review
Source: PLOS Glob Public Health. 2026 Sep 24;6(9):e0007080. doi: 10.1371/journal.pgph.0007080 (PMC13606984; doi:10.1371/journal.pgph.0007080)
Supplement: Supplementary file 5 [file pgph.0007080.s006.docx]

24 August 2026

Dear Editor,

Below is a document that describes our responses to the comments raised by the reviewers.

Felix Masiye

Corresponding author

**Point-by-point reviewers’ comments and authors’ answers**

Title of the manuscript: “Supply, demand and use of economic evidence in vaccine policymaking in Africa:

A scoping review”

| **#** | **Editorial and reviewers’ comments** | **Authors’ response** | **Line & page No.** |
| --- | --- | --- | --- |
| **#** | **Reviewer 1 comments** | **Authors’ response** |  |
| **1** | The authors have done a good job of addressing the comments. However, some comments were not addressed as they were mentioned as out of scope and so these should be reflected in limitations. First, the authors state that they did not do any critical assessment of the articles or check how the articles met the CHEERS checklist. This means that that authors cannot speak to quality of the evidence they reviewed, and this seems a limitation. | Thanks for the comment. We have included the following text in the limitations section.  “This being a scoping review, we did not conduct a quality assessment of the included articles so we cannot comment on the quality of the evidence.” | Page 31, Lines 625-626 |
| **2** | Also, they mention that they looked at the first and last author only and not the full author list, and so this needs to be clarified in the discussion and may mask collaboration with government coauthors and capacity strengthening through the process. | Thank you for the comment. We have included relevant information in the limitations section as below.  “Our analysis of authorship was limited to the first and last authors of each publication. This approach may have overlooked important collaborations and contributions from middle authors and participating institutions. However, we adopted this strategy because first and last authorship positions are generally regarded as the most influential in health research.” | Page 31, Lines 626-630 |
| **3** | The discussion section remains very long and would be helpful for it to be reduced and more focused as the reader gets lost on key messages due to its length. | Thank you for the comment. The discussion section seems long because we tried to address the different focus areas of the review which are pertinent given the scope of the review. We believe that this may be of value to the readership. | NA |
