# Supplementary material for: Supply, demand and use of economic evidence in vaccine policymaking in Africa: A scoping review
Source: PLOS Glob Public Health. 2026 Sep 24;6(9):e0007080. doi: 10.1371/journal.pgph.0007080 (PMC13606984; doi:10.1371/journal.pgph.0007080)
Supplement: Peer review history

# Peer review: Decision Letter 0

**DOI:** 10.1371/journal.pgph.0007080.r001

**Authors:** Katherine Kokkinias

20 May 2026

PGPH-D-26-00345

Economic evaluation in vaccine policy-making in Africa: A scoping review

PLOS Global Public Health

Dear Dr. Masiye,

Thank you for submitting your manuscript to PLOS Global Public Health. After careful consideration, we feel that it has merit but does not fully meet PLOS Global Public Health’s publication criteria as it currently stands. Therefore, we invite you to submit a revised version of the manuscript that addresses the points raised during the review process.

==============================

The manuscript has been evaluated by two reviewers, and their comments are available below.

The reviewers have raised a number of concerns that need attention. They request additional information on methodological aspects of the study to improve methodological clarity. They recommend modifications to the introduction and expansion of the analytical aspects of this study. They also suggest tempering conclusions to ensure that are supported by the data.

Could you please revise the manuscript to carefully address the concerns raised?

==============================

Please submit your revised manuscript by Jul 18 2026 11:59PM. If you will need more time than this to complete your revisions, please reply to this message or contact the journal office at globalpubhealth@plos.org. When you're ready to submit your revision, log on to https://www.editorialmanager.com/pgph/ and select the 'Submissions Needing Revision' folder to locate your manuscript file.

Please include the following items when submitting your revised manuscript:

- A letter that responds to each point raised by the editor and reviewer(s). You should upload this letter as a separate file labeled 'Response to Reviewers'.
- A marked-up copy of your manuscript that highlights changes made to the original version. You should upload this as a separate file labeled 'Revised Manuscript with Track Changes'.
- An unmarked version of your revised paper without tracked changes. You should upload this as a separate file labeled 'Manuscript'.

Guidelines for resubmitting your figure files are available below the reviewer comments at the end of this letter.

As the corresponding author, your ORCID iD is verified in the submission system and will appear in the published article. PLOS supports the use of ORCID, and we encourage all coauthors to register for an ORCID iD and use it as well. Please encourage your coauthors to verify their ORCID iD within the submission system before final acceptance, as unverified ORCID iDs will not appear in the published article. *Only*  the individual author can complete the verification step; PLOS staff *cannot*  verify ORCID iDs on behalf of authors.

We look forward to receiving your revised manuscript.

Kind regards,

Katherine Demi Kokkinias, Ph.D.

Staff Editor

PLOS Global Public Health

**Journal Requirements:**

1. Please amend your detailed Financial Disclosure statement. This is published with the article. It must therefore be completed in full sentences and contain the exact wording you wish to be published.

i. Please clarify all sources of financial support for your study. List the grants, grant numbers, and organizations that funded your study, including funding received from your institution. Please note that suppliers of material support, including research materials, should be recognized in the Acknowledgements section rather than in the Financial Disclosure.

ii. State the initials, alongside each funding source, of each author to receive each grant. For example: "This work was supported by the National Institutes of Health (####### to AM; ###### to CJ) and the National Science Foundation (###### to AM)."

iii. State what role the funders took in the study. If the funders had no role in your study, please state: “The funders had no role in study design, data collection and analysis, decision to publish, or preparation of the manuscript.”

iv. If any authors received a salary from any of your funders, please state which authors and which funders.

2. We note that your Data Availability Statement is currently as follows: All data used in the analysis have been provided as part of the submitted article.

Please confirm at this time whether or not your submission contains all raw data required to replicate the results of your study. Authors must share the “minimal data set” for their submission. PLOS defines the minimal data set to consist of the data required to replicate all study findings reported in the article, as well as related metadata and methods (https://journals.plos.org/plosone/s/data-availability#loc-minimal-data-set-definition).

For example, authors should submit the following data:

- The values behind the means, standard deviations and other measures reported;

- The values used to build graphs;

- The points extracted from images for analysis.

Authors do not need to submit their entire data set if only a portion of the data was used in the reported study.

If your submission does not contain these data, please either upload them as Supporting Information files or deposit them to a stable, public repository and provide us with the relevant URLs, DOIs, or accession numbers. For a list of recommended repositories, please see https://journals.plos.org/plosone/s/recommended-repositories.

If there are ethical or legal restrictions on sharing a de-identified data set, please explain them in detail (e.g., data contain potentially sensitive information, data are owned by a third-party organization, etc.) and who has imposed them (e.g., an ethics committee). Please also provide contact information for a data access committee, ethics committee, or other institutional body to which data requests may be sent. If data are owned by a third party, please indicate how others may request data access.

3. Some material included in your submission may be copyrighted. According to PLOS’s copyright policy, authors who use figures or other material (e.g., graphics, clipart, maps) from another author or copyright holder must demonstrate or obtain permission to publish this material under the Creative Commons Attribution 4.0 International (CC BY 4.0) License used by PLOS journals. Please closely review the details of PLOS’s copyright requirements here: PLOS Licenses and Copyright. If you need to request permissions from a copyright holder, you may use PLOS's Copyright Content Permission form.

Please respond directly to this email or email the journal office and provide any known details concerning your material's license terms and permissions required for reuse, even if you have not yet obtained copyright permissions or are unsure of your material's copyright compatibility.

Potential Copyright Issues:

a. Figure 2: please (a) provide a direct link to the base layer of the map (i.e., the country or region border shape) and ensure this is also included in the figure legend; and (b) provide a link to the terms of use / license information for the base layer image or shapefile. We cannot publish proprietary or copyrighted maps (e.g. Google Maps, Mapquest) and the terms of use for your map base layer must be compatible with our CC-BY 4.0 license.

Note: if you created the map in a software program like R or ArcGIS, please locate and indicate the source of the basemap shapefile onto which data has been plotted.

If your map was obtained from a copyrighted source please amend the figure so that the base map used is from an openly available source. Alternatively, please provide explicit written permission from the copyright holder granting you the right to publish the material under our CC-BY 4.0 license.

Please note that the following CC BY licenses are compatible with PLOS license: CC BY 4.0, CC BY 2.0 and CC BY 3.0, meanwhile such licenses as CC BY-ND 3.0 and others are not compatible due to additional restrictions.

If you are unsure whether you can use a map or not, please do reach out and we will be able to help you. The following websites are good examples of where you can source open access or public domain maps:

* U.S. Geological Survey (USGS) - All maps are in the public domain. (http://www.usgs.gov)

* PlaniGlobe - All maps are published under a Creative Commons license so please cite “PlaniGlobe, http://www.planiglobe.com, CC BY 2.0” in the image credit after the caption. (http://www.planiglobe.com/?lang=enl)

* Natural Earth - All maps are public domain. (http://www.naturalearthdata.com/about/terms-of-use/)

If the reviewer comments include a recommendation to cite specific previously published works, please review and evaluate these publications to determine whether they are relevant and should be cited. There is no requirement to cite these works unless the editor has indicated otherwise.

**Additional Editor Comments (if provided):**

[Note: HTML markup is below. Please do not edit.]

Reviewers' comments:

Reviewer's Responses to Questions

**Comments to the Author**

1. Does this manuscript meet PLOS Global Public Health’s publication criteria?

Reviewer #1: Yes

Reviewer #2: Partly

**********

2. Has the statistical analysis been performed appropriately and rigorously?

Reviewer #1: N/A

Reviewer #2: Yes

**********

3. Have the authors made all data underlying the findings in their manuscript fully available (please refer to the Data Availability Statement at the start of the manuscript PDF file)?

The PLOS Data policy

Reviewer #1: Yes

Reviewer #2: Yes

**********

4. Is the manuscript presented in an intelligible fashion and written in standard English?

Reviewer #1: Yes

Reviewer #2: Yes

**********

**Reviewer #1:** Introduction

The introduction is not very aligned with the focus of the review and should be rewritten. It is not clear there is a direct linkage between disease burden and generation or use of evidence for decision making. If there is, the authors have not made it clear.

The authors have not adequately captured other vaccine-related coping reviews that exist. There are some that are included in their selected articles but these should be mentioned in the introduction and it should be clear on how this review builds off the prior review and what the limitations of the vaccine costing or CEA reviews done to date are.

The introduction has some statements that seem subjective. For example, the statement on page 3 that reads “Currently, little is known about how immunization programs prioritize new vaccine introductions and vaccine delivery strategies.” seems subjective. We know prioritization is done at the global level through WHO and Gavi and some countries also have NITAGs to inform the introduction decision. Please provide validation for your statement here.

Methods

On page 4, what do the authors mean when they said they included studies of all study designs? Later in the PRISMA flow chart, there is mention that 7 studies were excluded because of wrong study design. This seems contradictory. Please explain what studies designs were in and out of scope.

The paragraph on page 6 that starts with “ we searched PubMed…” duplicates what was stated on page 4 last paragraph.

The selection of sources of evidence needs to be more specific. Can you provide initials of the authors who were involved in the steps included such as assessing abstracts, full text and also resolving conflicts of classification.

In the section on Data Items, please separate data items related to costing from those related to CEAs as these are different.

The authors mention that they asses “…existing structures utilizing economic evidence for decision making, challenges and enablers of economic evidence use for decision making…”. How was this done? This is one specific objective of this manuscript, but it is not clear how this information was assessed as the manuscripts were being reviewed.

Findings

Can the authors provide more information on why articles were excluded from the reviews. This is especially true for the 4037 studies screened, why were 3821 excluded. Similarity, of those selected for retrieval, why were 7 not retrieved?

As mentioned before, the methods say all study designs were included but the flow chart shows 7 were rejected for wrong study design. Please clarify.

Page 9 mentions study participants, should this be target population?

May you also add information on whether the studies had local collaborators and if any of the authors were government staff.

For table 3, I would suggest the authors separate costing studies from CEAs as the data extraction information is different. It would remove the blanks that are in the table.

Also, there is no clear order for Table 3 as it is neither alphabetical (by author, country) and not y year of publication. Please revise the table for better ordering.

In Table 3, why do you include the column on CEA threshold decision used and threshold applied as these are very similar.

The table seems to include reviews as well. Some information seems incorrect. For example, there is an article attributed to Slavkovsky et al. 2024 and mentions the country focus as Ethiopia, Rwanda and Uganda. But that article was a systematic review. Can you also identify the other review included in your extraction and whether the actual source articles reviewed are included.

The map on page 15 is not very informative. It could be improved by stating within each country, how many studies were done.

I am surprised to see NGOs such as PATH mentioned as funders as these do not fund studies.

Table 4 is hard to follow and understand.

You provide a synthesis of the CEAs but there is no similar synthesis for costing studies. What is the rationale for this?

How did the authors determine whether the studies informed policy?

There is mention on page 22 of qualitative studies. Where any insights provided from the quantitative studies on the use of evidence for decision making? What criteria or information were you looking for in the CEAs or costing studies that gave insights into whether the evidence generated was used for decision making?

Did the authors do a critical appraisal of the quality of the evidence?

Also was there a review of how the studies adhered to costing or CEA reporting standards such as CHEERs?

Discussion

Statements such as “It is hard for policy makers to rely on evidence from other settings for decision making...” seem subjective. This review does not seem to have done a critical appraisal of the existing evidence and so some of these statements made in the discussion are hard to justify,

There are several other subjective statements in the discussion section. An example is on page 23 which reads “Economic evidence has not been formally adopted as a requirement for vaccine policy in Africa”. This seems subjective as there are some countries that are requiring evidence as part of UHC. Also some NITAGs do use this evidence for decision making.

There is also an allusion that “…provided countries with free vaccines…”. There are no free vaccines as countries always have copayments and with Gavi 6.0 the payments are now even more.

Also Gavi through the VIS process had used evidence from global CEAs for decision making.

There were also several initiatives such as PROVAC to inform vaccine introduction decisions that should be acknowledged. That PROVAC work lead further development of models such as UNIVAC to enable CEAs. All these efforts have not been acknowledged by the authors and yet it is relevant context.

There is also lack of mention of the lack of country specific CEA thresholds and the lack of cross vaccine CEAs as most CEAs compare vaccine to no vaccine.

**Reviewer #2:**  This is a strong and very relevant topic, especially given the current push for domestic financing of immunisation programmes in Africa. The study is well executed from a scoping review perspective, and the dataset is solid.

That said, the paper reads more like a descriptive catalogue than an analytical piece. It shows what exists, but doesn’t go far enough in interpreting what that means or how it connects to real policy decisions. With some strengthening, it could make a much stronger contribution.

1. Strengthen the overall framing. At the moment, the paper feels like a compilation of studies rather than a clear argument. You present the evidence well, but it’s not organised around a strong central lens. It would help to structure the analysis more clearly, for example by thinking in terms of the supply of economic evidence, the demand for it, and how systems actually use or fail to use it. That would give the paper more direction.

2. The section on use of evidence needs more depth. This is probably the most important part of the paper, but it is currently quite thin. You note that economic evidence is used in a limited way, mostly for costing and planning. But the paper doesn’t really unpack how decisions are made in practice, or the role that different actors, especially donors versus governments, play in shaping those decisions. This section needs to go further and give a clearer picture of what is actually happening on the ground.

3. Clarify the link to policy-making. There is a bit of a mismatch between the title and the content. The title suggests a focus on policy-making, but most of the analysis is about the availability of studies. It would help to be more explicit about the difference between evidence existing, evidence being used, and evidence actually influencing decisions. If the conclusion is that influence is limited, that should be stated clearly and framed as a key finding.

4. Move beyond description and add interpretation. The results are detailed, but they often stop at reporting patterns without explaining them. For example, HPV and rotavirus dominate the literature, but the paper doesn’t explore why. Similarly, the concentration of studies in a few countries is noted but not really interpreted. Throughout the results section, you can strengthen the paper by briefly explaining what these patterns likely mean.

5. Strengthen the discussion on cost-effectiveness thresholds. You correctly highlight the widespread use of the 1–3 times GDP per capita threshold, but the discussion doesn’t really engage with the ongoing debate around this. It would help to briefly touch on issues like affordability versus cost-effectiveness, and the shift towards opportunity cost-based thresholds. This is an important part of the economic evaluation discussion and would strengthen the paper.

6. Make the policy implications more concrete. The discussion identifies important barriers such as limited funding, weak data systems, and lack of institutional structures. However, the recommendations remain quite general.

It would improve the paper to be more specific about what should change, for example in terms of institutionalising HTA, strengthening data systems, or aligning donor processes with national systems.

7. Simplify and better synthesise the ICER findings. The ICER results are detailed but not easy to interpret at a glance. The key messages are there, but they are buried in the tables. In the text, it would help to clearly state the main takeaways, such as which vaccines consistently show strong value for money, and how results change when Gavi support is included.

8. Minor Comments: There are a few small issues to clean up. For example, “Meta-Analyse” should be corrected to “Meta-Analysis.” Some sentences are quite long and could be tightened for clarity. The rationale for excluding COVID-19 vaccines could also be stated more clearly. The large tables are useful but might be better placed in the supplementary materials, with only key summaries in the main text. It would also help to keep terminology consistent throughout the paper.

Overall, this is a good paper with a strong foundation. The main issue is not the data, but the depth of analysis. With a clearer framing, stronger interpretation, and more direct policy relevance, it could be a very solid contribution.

**********

what does this mean?). If published, this will include your full peer review and any attached files.

**Do you want your identity to be public for this peer review?**  If you choose “no”, your identity will remain anonymous but your review may still be made public.

For information about this choice, including consent withdrawal, please see our Privacy Policy

Reviewer #1: No

Reviewer #2: **Yes:** Moses Chikoti Simuyemba

**********

[NOTE: If reviewer comments were submitted as an attachment file, they will be attached to this email and accessible via the submission site. Please log into your account, locate the manuscript record, and check for the action link "View Attachments". If this link does not appear, there are no attachment files.]

**Figure Resubmissions:**

# Author comment: Author response to Decision Letter 1

**DOI:** 10.1371/journal.pgph.0007080.r002

2 Aug 2026

Attachment Submitted filename: Response to reviewer comments_02.08.2028.docx

# Peer review: Decision Letter 1

**DOI:** 10.1371/journal.pgph.0007080.r003

**Authors:** Abram Wagner

21 Aug 2026

PGPH-D-26-00345R1

Supply, demand and use of economic evidence in vaccine policymaking in Africa: A scoping review

PLOS Global Public Health

Dear Dr. Masiye,

Thank you for submitting your manuscript to PLOS Global Public Health. After careful consideration, we feel that it has merit but does not fully meet PLOS Global Public Health’s publication criteria as it currently stands. Therefore, we invite you to submit a revised version of the manuscript that addresses the points raised during the review process.

The one reviewer still had a couple of comments - I think these could be addressed as added limitations. I am less concerned about the length of your discussion.

Please submit your revised manuscript by Sep 20 2026 11:59PM. If you will need more time than this to complete your revisions, please reply to this message or contact the journal office at globalpubhealth@plos.org. When you're ready to submit your revision, log on to https://www.editorialmanager.com/pgph/ and select the 'Submissions Needing Revision' folder to locate your manuscript file.

Please include the following items when submitting your revised manuscript:

- A letter that responds to each point raised by the editor and reviewer(s). You should upload this letter as a separate file labeled 'Response to Reviewers'.
- A marked-up copy of your manuscript that highlights changes made to the original version. You should upload this as a separate file labeled 'Revised Manuscript with Track Changes'.
- An unmarked version of your revised paper without tracked changes. You should upload this as a separate file labeled 'Manuscript'.

Guidelines for resubmitting your figure files are available below the reviewer comments at the end of this letter.

As the corresponding author, your ORCID iD is verified in the submission system and will appear in the published article. PLOS supports the use of ORCID, and we encourage all coauthors to register for an ORCID iD and use it as well. Please encourage your coauthors to verify their ORCID iD within the submission system before final acceptance, as unverified ORCID iDs will not appear in the published article. *Only*  the individual author can complete the verification step; PLOS staff *cannot*  verify ORCID iDs on behalf of authors.

We look forward to receiving your revised manuscript.

Kind regards,

Abram L. Wagner, PhD, MPH

Academic Editor

PLOS Global Public Health

**Journal Requirements:**

If the reviewer comments include a recommendation to cite specific previously published works, please review and evaluate these publications to determine whether they are relevant and should be cited. There is no requirement to cite these works unless the editor has indicated otherwise.

Please review your reference list to ensure that it is complete and correct. If you have cited papers that have been retracted, please include the rationale for doing so in the manuscript text, or remove these references and replace them with relevant current references. Any changes to the reference list should be mentioned in the rebuttal letter that accompanies your revised manuscript. If you need to cite a retracted article, indicate the article’s retracted status in the References list and also include a citation and full reference for the retraction notice.

**Additional Editor Comments (if provided):**

[Note: HTML markup is below. Please do not edit.]

Reviewers' comments:

Reviewer's Responses to Questions

**Comments to the Author**

Reviewer #1: (No Response)

**********

publication criteria?

Reviewer #1: (No Response)

**********

3. Has the statistical analysis been performed appropriately and rigorously?

Reviewer #1: (No Response)

**********

4. Have the authors made all data underlying the findings in their manuscript fully available (please refer to the Data Availability Statement at the start of the manuscript PDF file)?

The PLOS Data policy

Reviewer #1: (No Response)

**********

5. Is the manuscript presented in an intelligible fashion and written in standard English?

Reviewer #1: (No Response)

**********

**Reviewer #1:** The authors have done a good job of addressing the comments. However some comments were not addressed as they were mentioned as out of scope and so these should be reflected in limitations.

First, the authors state that they did not do any critical assessment of the articles or check how the articles met the CHEERS checklist. This means that that authors cannot speak to quality of the evidence they reviewed and this seems a limitation.

Also, they mention that they looked at the first and last author only and not the full author list, and so this needs to be clarified in the discussion and may mask collaboration with government coauthors and capacity strengthening through the process.

The discussion section remains very long and would be helpful for it to be reduced and more focused as the reader get's lost on key messages due to its length.

**********

what does this mean?). If published, this will include your full peer review and any attached files.

**Do you want your identity to be public for this peer review?**  If you choose “no”, your identity will remain anonymous but your review may still be made public.

For information about this choice, including consent withdrawal, please see our Privacy Policy

Reviewer #1: No

**********

[NOTE: If reviewer comments were submitted as an attachment file, they will be attached to this email and accessible via the submission site. Please log into your account, locate the manuscript record, and check for the action link "View Attachments". If this link does not appear, there are no attachment files.]

**Figure Resubmissions:**

# Author comment: Author response to Decision Letter 2

**DOI:** 10.1371/journal.pgph.0007080.r004

24 Aug 2026

Attachment Submitted filename: Response to reviewer comments 24082026.docx

# Editor report: Decision Letter 2

**DOI:** 10.1371/journal.pgph.0007080.r005

**Authors:** Abram Wagner

30 Aug 2026

Supply, demand and use of economic evidence in vaccine policymaking in Africa: A scoping review

PGPH-D-26-00345R2

Dear Professor Masiye,

We are pleased to inform you that your manuscript 'Supply, demand and use of economic evidence in vaccine policymaking in Africa: A scoping review' has been provisionally accepted for publication in PLOS Global Public Health.

Before your manuscript can be formally accepted you will need to complete some formatting changes, which you will receive in a follow up email. A member of our team will be in touch with a set of requests.

Please note that your manuscript will not be scheduled for publication until you have made the required changes, so a swift response is appreciated.

IMPORTANT: The editorial review process is now complete. PLOS will only permit corrections to spelling, formatting or significant scientific errors from this point onwards. Requests for major changes, or any which affect the scientific understanding of your work, will cause delays to the publication date of your manuscript.

If your institution or institutions have a press office, please notify them about your upcoming paper to help maximize its impact. If they'll be preparing press materials, please inform our press team as soon as possible -- no later than 48 hours after receiving the formal acceptance. Your manuscript will remain under strict press embargo until 2 pm Eastern Time on the date of publication. For more information, please contact globalpubhealth@plos.org.

Thank you again for supporting Open Access publishing; we are looking forward to publishing your work in PLOS Global Public Health.

Best regards,

Abram L. Wagner, PhD, MPH

Academic Editor

PLOS Global Public Health

***********************************************************

Reviewer Comments (if any, and for reference):
